# Smartphone-Based Markerless Motion Capture for Accessible Rehabilitation: A Computer Vision Study

**DOI:** 10.3390/s25175428

**Published:** 2025-09-02

**Authors:** Bruno Cunha, José Maçães, Ivone Amorim

**Affiliations:** 1 CINTESIS@RISE, CINTESIS.UPT, Department of Science and Technology, Portucalense University, Rua Dr. António Bernardino de Almeida 541, 4200-072 Porto, Portugal; 2 Porto Research, Technology & Innovation Center, Polytechnic of Porto (IPP), Rua Arquitecto Lobão Vital, 172, 4200-375 Porto, Portugal; ifa@isep.ipp.pt; 3 FEUP—Faculty of Engineering, University of Porto, Rua Dr. Roberto Frias, 4200-465 Porto, Portugal

**Keywords:** rehabilitation, computer vision, artificial intelligence, accessibility, machine learning

## Abstract

Physical rehabilitation is crucial for injury recovery, offering pain relief and faster healing. However, traditional methods rely heavily on in-person professional feedback, which can be time-consuming, expensive, and prone to human error, limiting accessibility and effectiveness. As a result, patients are often encouraged to perform exercises at home; however, due to the lack of professional guidance, motivation dwindles and adherence becomes a challenge. To address this, this paper proposes a smartphone-based solution that enables patients to receive exercise feedback independently. This paper reviews current Computer Vision systems for assessing rehabilitation exercises and introduces an intelligent system designed to assist patients in their recovery. Our proposed system uses motion tracking based on Computer Vision, analyzing videos recorded with a smartphone. With accessibility as a priority, the system is evaluated against the advanced Qualysis Motion Capture System using a dataset labeled by expert physicians. The framework focuses on human pose detection and movement quality assessment, aiming to reduce recovery times, minimize human error, and make rehabilitation more accessible. This proof-of-concept study was conducted as a pilot evaluation involving 15 participants, consistent with earlier work in the field, and serves to assess feasibility before scaling to larger datasets. This innovative approach has the potential to transform rehabilitation, providing accurate feedback and support to patients without the need for in-person supervision or specialized equipment.

## 1. Introduction

Rehabilitation plays a vital role in injury recovery and in restoring the physical capabilities of individuals who have disabilities or other health conditions. Traditional rehabilitation approaches often rely on in-person sessions supervised by healthcare professionals, which can be costly, time-consuming, and may lack accessibility for certain populations [1]. Since 2020, there has been a growing interest in integrating advanced technologies to augment rehabilitation processes and improve patient outcomes [2]. One area of technology that holds significant promise in the field of rehabilitation is Computer Vision (CV). CV allows computers to interpret visual data through image processing and Machine Learning algorithms. By capturing and analyzing the movements of individuals, these systems should be able to provide feedback without depending on the availability of a healthcare professional. They have the potential to revolutionize rehabilitation by enabling increased access to rehabilitation services.

In this paper, we focus on the application of CV for accessible physical rehabilitation. To accomplish this, we explore the use of markerless motion tracking techniques, which eliminate the need for external sensors or physical markers. This paper analyzes the key stages of markerless Computer Vision rehabilitation systems (data acquisition, pose estimation, and motion assessment) and introduces a smartphone-based prototype built for accessible home use.

The motivation behind this research comes from recognizing the significant challenges and limitations faced by individuals who require rehabilitation services. Simply put, rehabilitation is not accessible enough in terms of time, money, and availability. Developing a CV rehabilitation system aims to overcome these barriers and make rehabilitation more accessible to a wider population. This approach empowers individuals to perform rehabilitation exercises at home, removing the need for specialized equipment or constant supervision. This affordability and autonomy can extend to individuals with limited access to healthcare facilities or those facing financial constraints. This work builds upon prior clinical collaborations and is intended as a pilot feasibility study, designed to assess whether smartphone-based markerless analysis is viable for rehabilitation assessment at a low cost. The chosen sample size reflects logistical constraints and is in line with our previous exploratory studies in the field, including Lopes et al. [3].

Overall, this paper aims to contribute to the growing body of knowledge in the field of CV systems for rehabilitation by developing a motion tracking system that can improve the accessibility of rehabilitation processes. Through our work, we hope to empower individuals to take control of their rehabilitation journey, enabling them to enhance their overall well-being.

The remaining sections of this paper are organized as follows: In Section 2, the state of the art and the literature review are detailed. In Section 3, the implementation of the solution is described. Section 4 presents and analyses the assessment of the system evaluation and the collected results. Section 5 contains the conclusion and identifies the directions for future works.

## 2. State of the Art

The advent of intelligent technology has sparked great interest in the field of rehabilitation. Its integration enables the creation of new systems that have the potential to improve the efficiency of rehabilitation by helping to ensure exercises are performed correctly. They can also provide personalized assessments and progress tracking, among other benefits. Furthermore, this integration could increase the accessibility of rehabilitation, as these intelligent technologies can be autonomous and not require continuous professional supervision. This section reviews the state-of-the-art on the application of CV to rehabilitation. Firstly, we present a historical overview of vision-based motion tracking systems for rehabilitation. After that, there is a literature review on the specific topic of motion tracking systems for intelligent rehabilitation.

### 2.1. Historical Overview

According to the review by Colyer et al. [4], the first attempts to build vision-based motion tracking systems for rehabilitation purposes were based on manual digitization, which consisted of manually localizing points of interest, most typically joints, in each sequential image. This process is very time-consuming, demanding significant effort from experts to identify and track the relevant anatomical landmarks accurately, and can be subject to human errors, introducing potential inaccuracies in the analysis of exercises. The inherent drawbacks served as motivation to search for automated solutions. Automatic systems aim to automate the process of point localization and tracking, alleviating the burden of manual intervention and minimizing human errors. They also offer the advantage of reducing the time required for exercise analysis.

The first automated approach was automatic marker-based motion tracking systems [4]. As the name suggests, these systems relied on the placement of passive markers on the subject’s body, in strategic places such as joints, to make it easier to detect the position of these points of interest. This can be used to deduce the pose of the subject. The main advantages of these systems are automation and all that comes with it, such as eliminating the need for human interference and consuming less time, and the accuracy they present. Despite that, the requirement for physical markers causes many drawbacks. Markers take time to place and prepare; they are difficult to place in their correct positions, and that placement can shift from day to day; as they are not completely fixed to a joint, their position can differ from the joint’s position; they limit and constrain the subject’s movements. These limitations were the driving force for the next step: exploring markerless solutions.

Nowadays, research has focused intensively on markerless motion tracking systems [5], as they present numerous advantages, namely not requiring preparation of the subject, not being invasive or constraining the subject, and being much more accessible [4]. Although Colyer et al. [4] provides a useful synthesis, additional works have explored hybrid and semi-automated approaches, including early use of inertial measurement units (IMUs) and stereo vision systems in rehabilitation monitoring. These alternative technologies offer distinct advantages and trade-offs in terms of cost, portability, and clinical adoption. In addition to vision-based systems, these other rehabilitation monitoring solutions have leveraged IMUs, stereo vision cameras, and structured-light depth sensors, which remain active areas of research despite higher equipment complexity or cost [6]. This direction allows for more intelligent methods to be used, namely CV methods, that can detect pose and assess motion quality comparatively. The next sections detail the current state of research on markerless motion tracking systems for intelligent rehabilitation.

### 2.2. Accessible Motion Tracking Systems for Intelligent Rehabilitation

**Figure 1:**
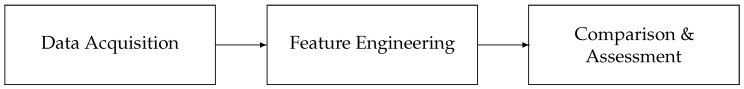
Typical structure.

In recent years, as this is an open research area, some motion tracking systems have been developed for intelligent rehabilitation for research purposes. Following the structure proposed by [7], in the domain of motion tracking systems for rehabilitation, systems are typically assembled in steps following a given flow. This flow tends to start with Data Acquisition, meaning the capture of a raw video of an exercise being performed by a given sensor. Then, *Feature Engineering* takes place, that is, meaningful features are extracted, and after that, these features are encoded, which is done by changing the way the features are represented. In this domain, the representation tends to be a time sequence of a group of joint positions or angles or other kinematic parameters. After that, there is a *Comparison and Assessment* step, where this sequence is compared to another, typically a reference or a desired sequence, in some way to derive an assessment of the quality of the performed exercise that will be the feedback the user will receive. This can be represented by the diagram in Figure 1.

We have identified and analyzed 13 scientific articles focusing on CV for rehabilitation, which are listed in Table 1. In this table we also categorize the articles based on their choices in terms of *Data Acquisition* methods, *Feature Engineering* techniques, and *Comparison and Assessment* methods. These 13 works were selected based on a structured review of English-language publications since 2018, using the terms ‘computer vision,’ ‘rehabilitation,’ and ‘motion tracking’ in article databases. The final selection focused on studies with explicit technical breakdowns of feature extraction and motion assessment. The articles that do not perform any of these steps or fail to mention it are under “Not specified”, and if those steps are performed but fall out of the scope of this review (i.e., the last step is not focused on rehabilitation), the cell will be marked “N/A”.    

In the following sections, we detail each step present in Figure 1 and analyze how it was addressed by each analyzed work. A commercial solution will also be presented.

### 2.3. Data Acquisition

From our analysis of existing literature on accessible technology, we observed that there are two main options for data acquisition: *Microsoft Kinect* and *RGB Cameras*.

*Microsoft Kinect* was widely adopted for CV research in rehabilitation. Numerous reviewed articles in this field have relied on *Microsoft Kinect* as their primary data collection tool [8,9,10,11,12]. After its introduction in 2010, *Microsoft Kinect* rapidly gained popularity, and one of the key factors contributing to its widespread adoption is its affordability, as it was a cost-effective solution for capturing RGB-D (colour and depth) images. Unlike traditional RGB cameras, besides colour and brightness information, *Microsoft Kinect* provides depth information for each pixel, which denotes the distance of that point from the sensor, according to [4]. *Microsoft Kinect*’s accuracy was reviewed and found to be superior to RGB-only systems [21]. The study conducted by Mousavi Hondori and Kademi deemed *Microsoft Kinect* acceptable for rehabilitation purposes, affirming its suitability as a reliable sensor in this domain. In addition to its low cost and effective RGB-D imaging capabilities, it is important to highlight that *Microsoft Kinect* also included an SDK. This SDK provided researchers with a comprehensive set of tools and libraries that facilitated the development of CV applications tailored explicitly for *Microsoft Kinect*. This made *Microsoft Kinect* much more valuable for researchers.

However, it is important to mention that *Microsoft Kinect* has drawbacks worth considering. These drawbacks include, but are not limited to, the following: first, Microsoft has discontinued production of the *Microsoft Kinect* [22]. This means that researchers are forced to switch to another device or go in a different direction. Additionally, although *Microsoft Kinect* was a low-cost solution compared to some alternative motion capture systems, it, as any other depth-sensor-based system, still represents an additional equipment requirement. Its utilization requires a dedicated *Microsoft Kinect* sensor, which moves away from the original goal of obtaining an as-accessible-as-possible solution.

The main alternative to *Microsoft Kinect* in the studied articles is the *RGB Camera*, which was the approach adopted by most of the reviewed articles [13,14,15,16,17,18,19]. In this set, we include standard, computer, and smartphone cameras, with the latter being the most accessible option.

Smartphones have become ubiquitous and readily available for a significant part of the population. Leveraging smartphones’ built-in cameras offers a more accessible approach to video capture, as the need for any extra equipment would not limit patients.

While the main limitation of an *RGB Camera* is its lack of precision compared to a system with a depth sensor, smartphone cameras have nonetheless been deemed suitable for clinical use [23]. In fact, Lam et al. [24] have concluded that smartphones will play a crucial role in the future of the application of CV for rehabilitation, contributing to accessibility. In Table 1, a summary of the different approaches for the Data Acquisition component is presented.

### 2.4. Feature Engineering

Feature engineering is the second step in the flow of a motion tracking system. It can encompass many smaller parts, including, but not limited to, feature extraction and feature encoding. Feature encoding is almost never detailed in the studied articles, as features are typically encoded as a time sequence. Thus, we will focus on feature extraction. Here, the CV task to be addressed is pose estimation. Typically, information deemed relevant is joint positions and angles. Obtaining this information involves tracking and extracting the spatial coordinates of key joints or measuring the angles between body segments during exercises. The extracted features are temporal sequences of the mentioned key points based on their evolution over time. Additionally, various measures can be incorporated, such as the range of an angle or the velocity of a joint, to provide more comprehensive information about movement dynamics.

Our study of the existing literature found that authors vary in their choice of tools for feature extraction. These choices include *OpenPose* [25], *BlazePose* [26], *OpenNI* [27], *FaceMesh*, *OpenFace*, *PoseNet* [28] and *Convolutional Pose Machines* [29].

When *Microsoft Kinect* is used for data acquisition, the provided SDK offers tools that allow developers to access pre-computed skeletal joint positions, orientations, and other relevant parameters. This means there is no need for explicit feature extraction. Therefore, almost all the studied articles that resort to *Microsoft Kinect* do not perform explicit feature extraction, making use of *Microsoft Kinect*’s SDK. The one exception is the work of Chen et al. [8], as they employ a custom process involving a series of image transformations using *OpenNI* [27] to extract spatial information, transforming this visual data into seven key points that they deem required for their rehabilitation system.

When it comes to raw images, feature extraction has to be performed. In the case of rehabilitation, as mentioned before, the task to be addressed is pose estimation. *OpenPose* [25] is the most popular choice, being used by Francisco and Rodrigues [14] and Ferrer-Mallol et al. [18], but it has the drawback of being computationally expensive, at least in comparison to other options, as will be seen in the next paragraph. Another pose estimation library, *BlazePose* [26], was employed by Yang et al. [16], offering similar results as *OpenPose* [25] but with significantly improved computational efficiency, making it apt to use with a smartphone. *BlazePose* [26] is favored for its rapid execution and is integrated into Google’s MediaPipe pose detector: OpenPose offers high accuracy but is computationally expensive, whereas BlazePose offers near-real-time performance suitable for mobile devices. More advanced feature encoding, such as dimensionality reduction or latent representations, remains underexplored in this context.

Abbas et al. [17] used *OpenFace* [30], a mobile-oriented facial behavior analysis toolkit. OpenFace focuses on head pose and facial expressions, making it valuable for rehabilitation assessments but limited to specific scenarios.

For real-time human pose estimation, Leechaikul and Charoenseang [15] employed *PoseNet* [28], a lightweight deep learning model. While it sacrifices some accuracy due to its lightweight nature, it serves the purpose efficiently. Li et al. [19] utilized *Convolutional Pose Machines* (CPMs) [29] in their work. CPMs leverage a Convolutional Neural Network (CNN) architecture with an iterative approach, generating heatmaps at each stage to estimate skeleton key points.

The fact that most reviewed articles used different tools showcases the diversity of the feature engineering domain, as can be seen in Table 1.

The present study focuses on positional coordinates, but future work could incorporate kinematic features such as velocity, acceleration, and movement smoothness to capture additional clinical nuances.

### 2.5. Comparison and Assessment

Once the relevant features, such as joint positions or angles, have been extracted and represented, they need to be compared and evaluated to assess the quality and effectiveness of the performed exercises. Feature comparison involves the analysis of the extracted features to determine how closely they align with reference movement patterns. Various techniques can be employed for feature comparison, including distance-based, model-less metrics, statistical-model-based metrics, or even Deep Learning. These approaches aim to quantify the similarity between the observed movement patterns and the desired ones.

Reviewing the existing literature on this topic, we can conclude that this portion of the process is the less standardized one, with most articles proposing their own version of a solution by combining several methods. That being said, one proposed option is a direct distance-based technique, Dynamic Time-Warping (DTW). However, most methods are model-based, be it via DL or by Hidden Markov Models (HMMs). Fuzzy logic is also present in more than one article.

Chen et al. [8] developed a vision-based rehabilitation system with a primary focus on action identification, discerning if a subject is performing a rehabilitation exercise and identifying the specific exercise. They employed DTW to measure the similarity between sequences of varying lengths, enabling comparison of different exercise paces. Their system achieved impressive results, boasting a 98.1% accuracy rate for action identification.

Su et al. [9] employed DTW for comparison and implemented an Adaptive Neuro-Fuzzy Inference System (ANFIS) for evaluation. ANFIS combines Neural Networks (NNs) and fuzzy logic principles. Their evaluation module includes a trajectory evaluator based on a Sugeno-type ANFIS, a speed evaluator, and an overall performance evaluator based on a Mamdani fuzzy inference model. The system matched physicians’ scores at an 80.1% rate, demonstrating its effectiveness.

Capecci et al. [10] devised an innovative approach to evaluate rehabilitation exercises using a Hidden Semi-Markov Model (HSMM). Unlike traditional HMMs, HSMM models state durations as distributions. Collaborating with clinicians, they trained the HSMM with data from the seven top-rated subjects. The HSMM calculates a comprehensive score based on observation likelihood. Through hyperparameter tuning, they optimized the HSMM to match clinician ratings. This resulted in a significant correlation for two of the five exercises. Compared to DTW, the HSMM outperformed DTW in correlating with clinician scores for four of the five exercises.

Ciabattoni et al. [13] collaborated with physiotherapists to establish target values, incorporating tolerances for five exercises. They designed a score function based on these targets and measured values for specific joint angles. The overall exercise score is computed as the average or maximum of individual scores. They used a Takagi-Sugeno Fuzzy Inference System to determine the subject’s global score, offering flexibility in weighting exercise targets according to physiotherapist recommendations.

Francisco and Rodrigues [14] utilize a Modular NN for exercise assessment. They begin by extracting and storing joint angles, focusing on four angles. After that, their system is divided into their detection module, which identifies what exercise is being performed, and their measure module, which employs an NN to assess exercise correctness. The initial network architecture was straightforward and comprised three layers. Multiple configurations were tested to obtain the best architecture and evaluated using accuracy and the area under the Receiver Operating Characteristic (ROC) curve. Ultimately, the most effective architecture ended up featuring eight hidden layers and achieving 94.54% accuracy and an area under the ROC curve ranging from 0.8 to 1, depending on the exercise.

Liao et al. [20] employ a Gaussian Mixture Model (GMM) log-likelihood performance metric for rehabilitation, leveraging its ability to effectively capture the inherent variability in human movements. This metric is incorporated into a Deep-Learning model comprised of a complex deep NN that includes convolutional layers, recurrent layers, and temporal pyramids. It outputs movement quality scores for input exercises. In their evaluation, they compared the GMM log-likelihood metric to Euclidean distance, Mahalanobis distance, and DTW distance, focusing on its capacity to distinguish correct from incorrect movements. The results showcased the superiority of the proposed metric, although DTW distance and Euclidean distance displayed noteworthy results. Additionally, they assessed their Deep-Learning model against other NN models, using absolute deviation as the performance metric. The model demonstrated overall superior performance, although specific models outperformed it in certain exercises within the dataset.

Table 1 also showcases the diversity in Comparison and Assessment methods. In summary, model-based approaches such as HMMs and neural networks offer flexibility and robustness but require large datasets. DTW, while simpler, is more interpretable and easier to deploy. The lack of standardization in this step reflects the diversity of rehabilitation exercises, absence of benchmarking datasets, and the tension between clinical interpretability and computational complexity.

### 2.6. Commercial Systems

In addition to research efforts, there are also commercial solutions available in the field of CV for rehabilitation. One such solution is Exer Health [31], a patient mobile app designed to enhance the rehabilitation process. Exer Health aims to keep patients engaged and gather critical health assessment data while they perform exercises at home. The app claims to measure range of motion, counts repetitions, recommends form adjustments, and provides real-time feedback to patients. This feedback helps patients adhere to their recovery protocols, and the data collected can be used by professionals to evaluate progress throughout the rehabilitation journey. Moreover, Exer Health offers professionals an intuitive mobile app that facilitates the creation of high-touch, closed-loop recovery protocols. This gives patients a richer experience while providers focus on delivering optimal care.

Regarding technology, Exer Health claims to employ a proprietary motion-AI platform that powers all of its digital health software. The platform is said to run “on the edge,” utilizing common laptops, phones, and tablets. This approach ensures accessibility and ease of use for patients and professionals, allowing seamless technology integration into rehabilitation. Although they do not give any details, they mention the use of NNs, Machine Learning, and CV.

**Figure 2:**
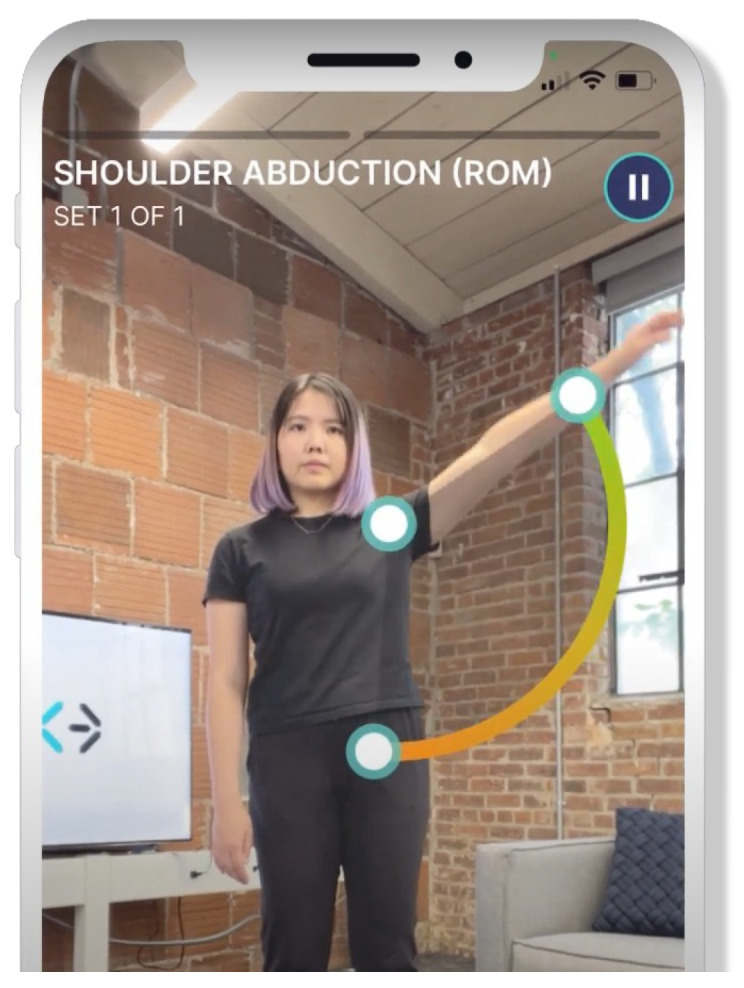
Exer Health App. Source: Exex Health [31].

Figure 2 shows an example of how the Exer Health application works.

The main problem with Exer’s solution is that they do not reveal much about it, with their explanations being very generic when it comes to the technology used and how they used it. Our work differs from theirs in that sense, as our objective is to make this research public and available for anyone to understand. On this topic, we would like to also highlight the mobile system presented by Pereira et al. [32]. Although it is in an early development stage, the proposed integration of intelligent analysis for exercise compression is very promising.

### 2.7. Available Datasets for Modeling Computer Vision Systems

The availability of high-quality and diverse datasets is very important in training CV models for rehabilitation applications. Whereas in other motion modeling applications based on CV, there is a wide range of large public datasets; in the case of rehabilitation data, authors tend to collect their own data, obtaining relatively small datasets [7]. In Table 2, we compiled the most relevant datasets found during our research.

However, these datasets all mention that their data is captured by an RGB-D camera such as *Microsoft Kinect*. This is also true for other datasets found during the literature review, as well as others that mention the use of body-worn sensors. The dominance of RGB-D datasets limits the applicability of models to RGB-only systems, which are more suitable for smartphone deployment. At present, there is a gap in public datasets for smartphone-based rehabilitation, which restricts external validation and hampers generalization across platforms.

### 2.8. Takeaways and Conclusions

The previous sections have explored the intersection of CV and intelligent rehabilitation systems, examining the relevant literature and gaining insights into this field.

We observed that data acquisition methods have evolved over time. While *Microsoft Kinect* was once a prominent choice, its discontinuation has led to the emergence of smartphone cameras as accessible and reliable alternatives for rehabilitation purposes.

In terms of feature engineering techniques, OpenPose is the most popular choice. Although it is widely used for pose estimation, it is computationally expensive, so we highlighted an alternative, BlazePose, that offers greater efficiency and speed.

The comparison and assessment of exercises involve diverse techniques, including DTW, fuzzy inference systems, HSMMs, and deep NNs. These approaches aim to quantify the similarity between observed and desired movement patterns. Every studied article mentioned satisfactory results.

What we conclude by comparing the different studies is that DTW seems to be a standard method. It could be interesting to use as an initial method and a term for comparison of different methods, serving as a benchmark. We also found that the trend is the use of DL, as in most fields of computer science. Fuzzy logic seems to be regarded as an effective tool for this task, as well as HMMs. We also discussed Exer Healt [31], a commercial solution with a promising premise. Unfortunately, they are not very transparent: they state that they employ a proprietary motion-AI platform, and we found mentions of NNs, Machine Learning, and CV, but no concrete details are given in terms of the specific techniques used, instead opting for very generic explanations. Another important aspect mentioned is the available datasets. As detailed before, there are not many large public datasets, which is something researchers have to deal with. Typically, the solution is for authors to collect their own data. Building on the insights from this review, our work takes the discontinuation of *Microsoft Kinect* into consideration and explores the potential benefits of smartphone cameras. While methods such as DTW are frequently used in research, they are not clinical standards. Deep Learning is increasingly applied in recent literature [2], but model transparency and data scarcity remain challenges. This study addresses the gap in evaluating smartphone-only, markerless rehabilitation systems and provides an open, reproducible benchmark for future work.

## 3. Implementation

### 3.1. Architecture

The proposed system follows the structure presented in Section 2, meaning it involves three components: Data Acquisition, Feature Engineering, and Comparison and Assessment. Therefore, we had to make technological and architectural choices for each one of these components. The following subsections will detail the choices made for each of the components. A smartphone camera was chosen due to its ubiquity and integration into daily life, ensuring feasibility for at-home rehabilitation use. BlazePose, part of Google’s MediaPipe framework, offers real-time pose estimation on mobile devices and has proven effective in similar tasks. DTW, a distance-based alignment technique, was selected for its simplicity and clinical interpretability, despite the availability of more complex model-based metrics.

#### 3.1.1. Data Acquisition: Smartphone Camera

In the Data Acquisition component, we had to choose between *Microsoft Kinect* and the RGB camera, as is explained in Section 2.3. Deriving from what is mentioned in the aforementioned section, we opted for the RGB camera, more specifically, the smartphone camera, due to the ubiquity of the smartphone, aligning with the focus on accessibility. Furthermore, it should allow for a more straightforward and user-friendly approach to data collection.

#### 3.1.2. Feature Engineering: MediaPipe’s Pose Landmarker (BlazePose)

Regarding Feature Engineering, the range of choices was much broader, as detailed in Section 2.4. The most popular choice, OpenPose, had the drawback of being computationally expensive, which could pose problems given the choice of the smartphone, a less computationally powerful device. One of the alternatives was BlazePose, which presented results similar to OpenPose but was much less computationally expensive, making it suitable for a smartphone. One framework that implements the BlazePose algorithm is the MediaPipe Pose Landmarker. MediaPipe [36,37] is an open-source framework created by Google that provides a toolkit for the development of Machine Learning applications, mainly focused on helping people design and implement solutions for vision, audio, and text-based Machine Learning tasks. In terms of audio and text, it provides developers with tools suitable for various classification, detection, and embedding tasks. When it comes to vision-based tasks, it offers tools to perform Object Detection, Image Classification, Hand Landmark, Hand Gesture Recognition, Image Segmentation, Interactive Segmentation, Face Detection, Face Landmark Detection, and the one we are interested in, Pose Landmark Detection.

**Figure 3:**
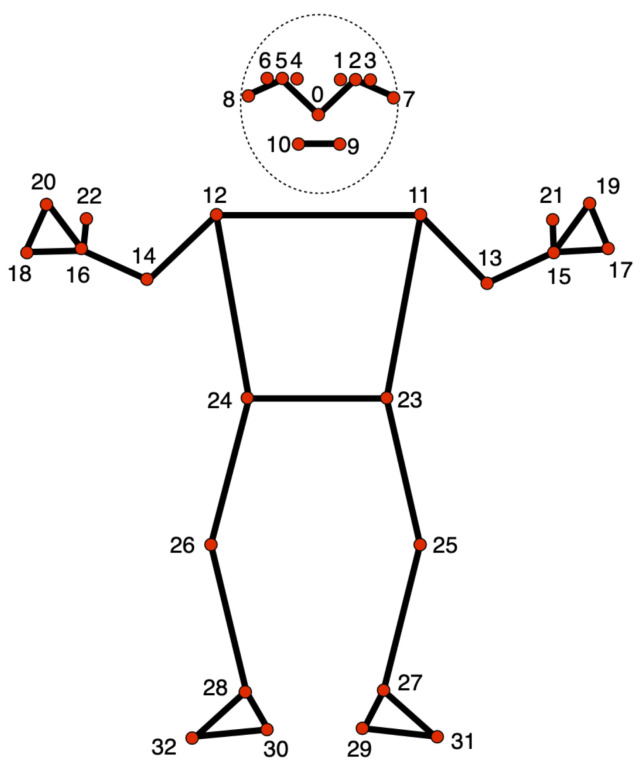
MediaPipe Pose Landmarker Model. Source: Google Mediapipe [38].

The MediaPipe Pose Landmarker [38] is a component within the broader MediaPipe framework designed for human pose estimation. This specific module is focused on identifying and tracking key points on a person’s body, enabling applications to access an estimate of the human pose. It receives as input still images, decoded video frames, or live video feed and outputs pose landmarks in normalized image coordinates or pose landmarks in world coordinates. MediaPipe includes a pose landmarker model that outputs 33 key landmarks. Figure 3 shows the position of these 33 landmarks in the human body.

**Figure 4:**
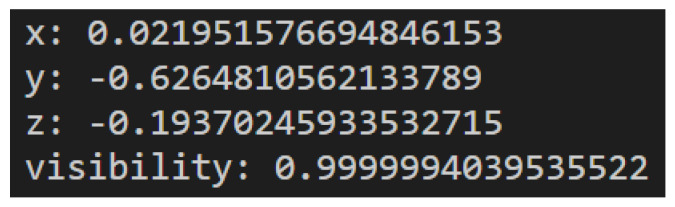
Example of landmark output.

The output of the MediaPipe Pose Landmarker contains X, Y, and Z coordinates for each landmark and a visibility factor, representing how likely it is that the landmark is visible. As mentioned before, the MediaPipe Pose Landmarker can output pose landmarks in either normalized image coordinates or world coordinates. If the output is normalized image coordinates, X and Y are normalized between 0 and 1 in relation to the image’s width and height. The Z coordinate is the landmark depth, with the hips as the origin. It has the same magnitude as X, and the smaller the value is, the closer it is to the camera. If the output is world coordinates, X, Y, and Z are real-world three-dimensional coordinates in meters, also with the hips as the origin. An example of the output of a landmark is presented in Figure 4.

#### 3.1.3. Comparison and Assessment: Dynamic Time Warping

To perform the Comparison and Assessment part of the proposed system, we opted for DTW. DTW is an algorithm used to assess how similar two time series are by measuring the distance between those two series. Let us use as an example two time series *A* and *B*. The simplest way to measure the distance between two time series would be to use Euclidean distance, which is computed as the square root of the sum of the squares of the differences between *A*_*i*_ and *B*_*i*_, where *A*_*i*_ represents the value of time series *A* at index *i* and *B*_*i*_ represents the value of time series *B* at the same index. Equation (1) introduces the Euclidean distance between the time series *A* and *B*.

(1) $$EuclideanDist=\sqrt{\sum_{i=1}^{n}{\left(A_{i}-B_{i}\right)}^{2}}$$

The problem with this measure is that it does not account for shifts along the time axis. For example, if two time series are equal but one of them is shifted in time, Euclidean distance would indicate a difference between both series.

For example, let us consider the following sequences: *A*^′^ = [0, 1, 2, 3, 0, 0] and *B*^′^ = [0, 0, 1, 2, 3, 0]. We can understand that the sequences represent the same evolution, but sequence *B*^′^ starts later than sequence *A*^′^. If we calculate the Euclidean distance between these series, we would obtain a result of $\sqrt{12}\approx 3.46$, when the desirable result would indicate a smaller difference.

With the aim of solving this problem, DTW was developed [39]. The idea behind DTW is the computation of a path that warps points of the two series to one another in order to obtain a more intuitive relationship between the series. Warping two points means creating a correspondence between them. This path includes every index of both time series, meaning that every point in one sequence is warped to a point in the other sequence. The goal is to compute the optimal warp path, i.e., the one with the minimum distance. To do so, we must first compute a two-dimensional distance matrix, *D*. Given the same time series *A* and *B*, *D* is of size |A|×|B|, where |A| and |B| are the lengths of *A* and *B*, respectively. Therefore, each axis represents one of the time series. The process of computing the matrix is done dynamically, starting with D(0,0) and finishing with D(|A|,|B|) The value of each cell in *D* is computed by the following formula:

(2) $$D\left(i,j\right)=Dist\left(A_{i},B_{j}\right)+\min\left[D\left(i-1,j\right),D\left(i,j-1\right),D\left(i-1,j-1\right)\right],$$

where i≤|A| and j≤|B|. In every calculation, we add the distance between the two points in each axis at the current index, *Dist*(*A*_*i*_, *B*_*j*_), to the minimum value of the three cells that precede the current one. This is because we know that the optimal path must pass through one of those cells and that one of them contains the minimum possible distance, so we add the current distance to the smallest of those three values. *Dist*(*A*_*i*_, *B*_*j*_) can be calculated by various distance measures, such as Euclidean distance or Manhattan distance. A comparative study between different distance measures for DTW showed that Euclidean distance obtains the best results [40]. After we fill the matrix, the value of the DTW distance between the two sequences is the value at D(|A|,|B|).

**Figure 5:**
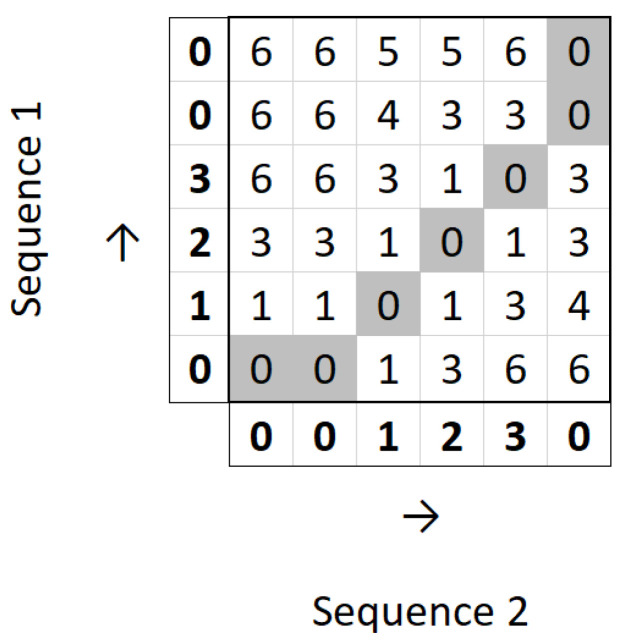
Distance matrix for DTW calculation.

For example, let us consider the same sequences *A*^′^ and *B*^′^ defined above. Figure 5 shows the filled distance matrix for the calculation of DTW between those sequences.

It can be observed that we obtain the optimal path from D(0,0) to $D(|A^{\prime }|,|B^{\prime }|)$ and are left with the value 0 at $D(|A^{\prime }|,|B^{\prime }|)$. This means that the DTW distance between the two sequences is 0.

The choice for DTW stems from the fact that it is simple, being a distance-based metric, but presents impressive results measuring the similarity between sequences of movements. And we also had scalability into account: the current prototype focuses on offline analysis but real-time execution is a key goal. MediaPipe inference operates at around 30 fps on modern smartphones, and future system iterations will leverage framewise feedback to support real-time movement correction.

### 3.2. Dataset

**Figure 6:**
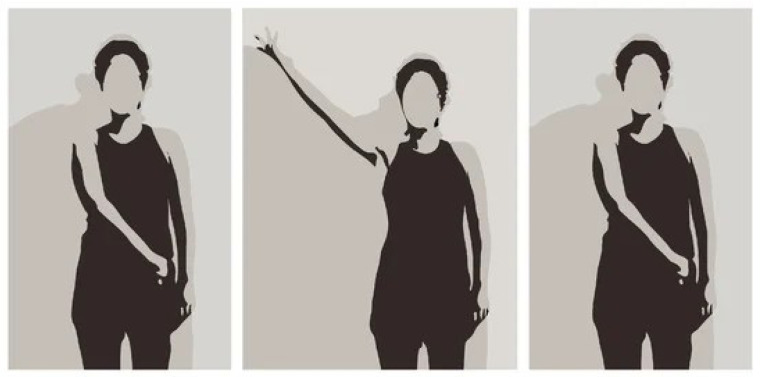
Frames of the *Diagonal* exercise. Source: Pereira et al. [32].

Collaborating with Escola Superior de Saúde (ESS) of the Instituto Politécnico do Porto was instrumental in accessing a pre-existing dataset of exercises; and, also, their healthcare professionals were involved in the selection of clinically relevant joints and validation of movement ranges based on their rehabilitation protocols. The data that is included was collected with the primary aim of examining rehabilitation exercises. Subsequently, it has been utilized in various works, including the study conducted by Lopes et al. [3]. Fifteen patients, labeled with IDs 1 to 15, were asked to perform two distinct exercises, *Diagonal* and *Rotation*, each repeated three times. The limited cohort size was a deliberate choice reflecting the pilot nature of this study. Our previous work with ESS with similar experimental constraints adopted comparable sample sizes (Lopes et al. [3]) and our goal was to assess technical viability and alignment with clinical scores before pursuing broader deployment or generalization. Those exercises were recorded with a smartphone camera, and those videos are what constitute our dataset, as well as two reference videos performed by healthcare professionals—one for each video. Figure 6 shows frames of a video from the dataset.

That is a total of 90 examples, 45 for each exercise, plus the two reference videos. The dataset has been evaluated by an advanced state-of-the-art system, the Qualisys Motion Capture System (QTM) [41], that can evaluate Range of Motion (ROM), being scored from 0 to 100. The specifics of the conversion from a QTM evaluation to such a score are further detailed in another publication by Lopes et al. [3]; but, in sum, it involves identifying key points of movement and measuring the maximum and minimum angles at these points. However, the reference scores are not attributed to each example in the dataset but to each patient, making a total of 30 scores: one for each of the two exercises performed by the 15 patients. This score represents a movement quality index based on comparing ROM between instances of subjects performing Guided Exercises (GEs) and Non-Guided Exercises (NGEs) and is calculated by the following equation:

(3) $$MovementQualityIndex\left(\%\right)=\left(1-\frac{ROM_{GE}-ROM_{NGE}}{ROM_{GE}}\right)\times 100$$

The selected dataset contains outliers, which have been explicitly identified by the physiotherapists responsible for its collection. Section 4.2 provides an in-depth exploration of the strategies employed to deal with these outliers. As a final note, it is important to notice that public datasets were considered; however, there was none that matched our criteria of RGB-only, smartphone-acquired, rehabilitation-specific data synchronized with QTM scores.

### 3.3. System Development

#### 3.3.1. Comparing Two Videos

In order to obtain a system that compares two videos, we devised the following plan:

1. Start by focusing on a single exercise and identify the relevant joints for that exercise.
2. Process one input video:
  (a) Read the video.
  (b) Extract values of relevant joint positions for each frame.
  (c) Create and save a sequence of these values.
3. Process another input video in the same fashion.
4. Calculate the DTW distance between two sequences.

Thus, we started by selecting one of the two mentioned exercises, *Diagonal* and *Rotation*. There were no substantial differentiating factors between the two, so we opted for *Diagonal*. We then wanted to identify which joints were relevant for the exercise. In order to do that in a clinically sound manner, we made use of our collaboration with Escola Superior de Saúde. We asked them which joints were relevant for comparing this exercise. They mentioned that they divided joints into three major groups for their comparison: Head, Trunk, and Shoulder. They also mentioned which landmarks of the MediaPipe Pose Landmarker Model should be included in each joint group. This meant that the relevant joints were organized as follows:

- Head: Landmarks 0 to 10.
- Trunk: Landmarks 11, 12, 23, 24.
- Shoulder: Landmarks 12, 14, 16.

The next step was to process an input video. This involved developing a Python script where videos could be processed, and relevant data could be retrieved, particularly the positions of the relevant landmarks for each frame. In order to process the video, we resort to the OpenCV library [42], which is an open-source software library designed for Computer Vision and Machine Learning applications. It offers a comprehensive set of image and video processing tools, including modules for image manipulation, object detection, feature extraction, and more. It is widely utilized in Computer Vision applications. We then made use of the MediaPipe Pose Landmarker to perform pose estimation and obtain the X, Y, and Z coordinates of each landmark present in the relevant joint groups for each frame. At this point, we faced a choice between using MediaPipe Pose Landmarker’s Landmarks or WorldLandmarks. We opted for WorldLandmarks because they are relative to the subject’s body. Landmarks are sensitive to the position of the subject in the image, which can cause problems when comparing two videos. These coordinates were saved after each frame, and we created a sequence of coordinates for each relevant landmark. Each sequence was essentially a list that had as elements smaller lists, one for each frame, that had as elements the coordinates of that landmark at that frame. After processing a video, we were left with a set of lists, one for each landmark, representing the evolution of the position of that landmark over time.

A generic sequence for a landmark would be represented as:

(4) $$\left[\left[x_{t=1},y_{t=1},z_{t=1}\right],\left[x_{t=2},y_{t=2},z_{t=2}\right],\ldots ,\left[x_{t=T},y_{t=T},z_{t=T}\right]\right]$$

In this sequence, each list represents the X, Y, and Z coordinates of the landmark for each frame. After we had processed two input videos and obtained the sequences that represented the motion in terms of the evolution of the position of the relevant joints, we were ready to compare them and assess how similar those sequences were and, consequently, how similar the exercises being performed were. To compare the two exercises, we opted to calculate the distance between each relevant landmark’s evolution in one video to the corresponding one in the other video and then sum up those distances according to the grouping suggested before, meaning the distances between landmarks in the head group would be summed up to obtain the overall distance measure for the head. In order to calculate the distance between two sequences, we used DTW.

There are many libraries that implement DTW in Python (version 3.10.11 used), as is the case of dtaidistance, TSLearn, or FastDTW. FastDTW is the most commonly used one [43], but it is actually an approximation, designed to perform the DTW calculation faster than the original algorithm [44], whereas both dtaidistance and TSLearn perform the full DTW algorithm.

However, it has been claimed that FastDTW might actually not be faster and, because it is an approximation, the trade-off between accuracy and speed might not be worth it [43].

In order to make a choice, we compared the execution of the three algorithms as well as a generic ad hoc DTW implementation. To compare the execution time of each algorithm, we ran each one on the same two videos ten times, registering the average of their execution times.

**Figure 7:**
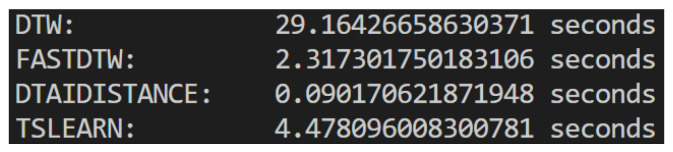
Comparison of times of execution for the different DTW implementations.

**Figure 8:**
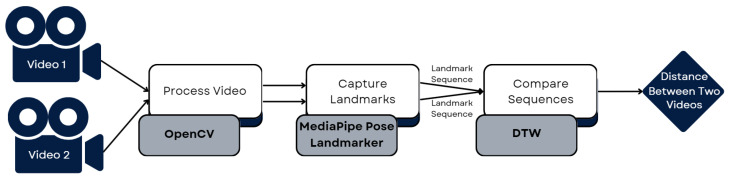
Pipeline for comparing 2 videos.

As we can see in Figure 7, *dtaidistance* had the smallest execution time, so that was the final choice. This meant we could now compare two videos and obtain distance measures for the three landmark groups, concluding the initial part of our development plan. Figure 8 shows the pipeline followed to develop the part of the system that compares two videos and obtains a distance between them. It takes two videos as input, processes them using OpenCV, captures the landmarks of both using the MediaPipe Pose Landmarker, obtaining the landmark time sequences, which are then compared using DTW, resulting in a measure of the distance between the two videos.

#### 3.3.2. Generalizing for the Whole Dataset

After we had built a system capable of comparing two videos and obtaining a measure of the difference between the videos, our intention was to generalize and obtain that measure for each exercise in the dataset. Thus, we wrote a Python script that would do this for us. First, it processed the reference video for one exercise and saved the landmark sequences. Then, it iterated through the dataset, reading each video of that exercise and obtaining the landmark sequences, comparing them, using DTW, to the reference. This gave us a distance score for the three landmark groups in each video, which were then saved in a data frame and, subsequently, in a CSV file. This process was repeated for the second exercise, leaving us with two files with the distances recorded between the time sequences of the reference exercise and each of the examples in the dataset. Each file was divided into distances for the Head, Trunk, and Shoulder landmark groups. These files were then used as input for the evaluation of the proposed system, comparing the results obtained to those obtained by the QTM mentioned in Section 3.2.

Table 3 shows a portion of the CSV file that contains the distances between the *Diagonal* exercise examples in the dataset and the *Diagonal* reference exercise, divided into the Head, Trunk, and Shoulder landmark groups.

**Figure 9:**
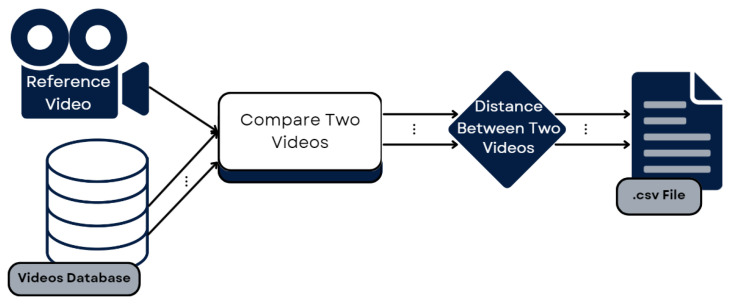
Pipeline for comparing the entire dataset to the reference.

Figure 9 shows the pipeline followed to develop the part of the system that iterates through the dataset, comparing every example video to the reference and obtaining a distance for each one.

This section details how the CV-based motion tracking system was implemented, including the architecture of the proposed system and the choices that were made at each step of the system flow, the dataset that was used and the technologies that were selected. While this study involved manual preprocessing steps, future iterations aim to integrate full automation of video segmentation, joint extraction, and scoring for scalability.

## 4. System Evaluation and Results

This section is focused on the evaluation of the proposed system, the presentation and discussion of the obtained results. The first section explains the concept of data normalization, why it is necessary, and how we opted to apply it in our data. After that comes a section on the details of the proposed system evaluation, where we discuss the metrics used for evaluation and how we handled outliers. Then, there is a section presenting the results and a following section discussing them. After that, there is a section on statistical analysis. Finally, there is a small summary of the section.

### 4.1. Data Normalization

To evaluate the performance of the proposed system, we will compare the results obtained by the QTM, mentioned in Section 3.2. However, the results obtained are a distance measure, whereas the dataset is labeled with scores on a scale of 0 to 100. To ensure a meaningful comparison, we need to make sure that both sets of results are on the same scale, which means we need to transform our distance measures into a 0 to 100 score. One way to achieve this is through data normalization.

Data normalization is a process in which data is scaled to a specific range. It is usually performed as a preprocessing step before the comparison of two sets of data and allows the comparison of data with different ranges. There are many different ways of performing data normalization, including, for example, Z-score normalization or min-max scaling.

Lima et al. [45] conducted a comprehensive study comparing various normalization options, more specifically for the normalization of time series. They mention that Z-score is by far the most popular option, with many authors not even considering the alternatives. It is particularly well-suited for normally distributed data. However, they conclude that it may not always be the best option and recommend at least exploring one more option before using just Z-score—more specifically, maximum absolute scaling.

Z-score normalization transforms data to have a mean of 0 and a standard deviation of 1, with each transformed value being calculated with the following equation, where *μ* is the mean of the data, and *σ* is the absolute deviation:

(5) $$x^{\prime }=\frac{x-\mu }{\sigma }$$

Min-max scaling scales the data to a specific range, often 0 to 1, but in our case, 0 to 100, based on specified minimum and maximum values. These values are usually the maximum and minimum values of the dataset, but we could select values that correspond to what would be considered, in the original scale, a score of 0 and a score of 100. For example, in our case, the minimum would be 0, as the ideal performance of an exercise corresponds to a distance of 0 when comparing the two time sequences, and the maximum would be the values obtained by comparing two very different videos. One crucial detail to consider is that, in our case, larger distances correspond to lower scores and vice versa, which means that, after scaling is done, we invert the scale by subtracting each score from 100. The transformed values are calculated via the following equation:

(6) $$x^{\prime }=\frac{x-min}{max-min}$$

Maximum absolute scaling works in a very similar way but just considers the maximum absolute value. Each value is obtained with the following equation.

(7) $$x^{\prime }=\frac{x}{\left|max\right|}$$

Because all our data is positive and we know the minimum value is 0, maximum absolute scaling and min-max scaling have the same behavior. From this point on, we will only refer to min-max scaling.

Before choosing the normalization technique, we must first analyze our data.

**Figure 10:**
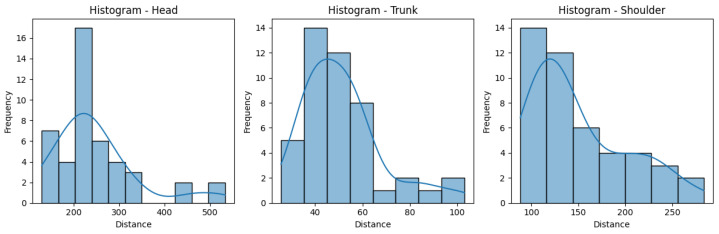
Histograms for the distances obtained for the *Rotation* exercise.

**Figure 11:**
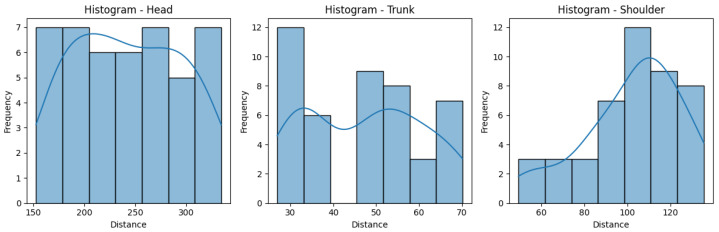
Histograms for the distances obtained for the *Diagonal* exercise.

Figure 10 and Figure 11 help us visualize the distribution of our data. They include histograms for the three joint groups for each exercise. Examining the presented graphs, it becomes evident that the distribution of the data does not have the characteristics of a normal distribution, as there are clear deviations from the expected bell-shaped curve. This tells us that the data does not conform to a normal distribution and that Z-score normalization would not necessarily be suitable for our data. However, due to its immense popularity, and given most authors will not even consider not using Z-score, we decided to compare the effectiveness of these normalization techniques, applying both Z-score and min-max scaling to our data. Finally, we calculate the average scores for three exercises per ID, providing an overall performance score for each exercise. This aggregated score is what we compare to the QTM score. An example of the aggregated scores can be seen in Table 4.

### 4.2. System Evaluation

The evaluation process is crucial for assessing the performance and practical utility of the system. A thorough evaluation can not only validate a system but also identify areas for improvement. As mentioned before, to evaluate the proposed system, we will compare the results obtained to those obtained by the QTM. However, it is important to clarify that, to the best of our knowledge, no other approaches utilize the same data structure as ours, which involves manually annotated QTM evaluations by physicians combined with a mediapipe/DTW intelligent analysis.

#### 4.2.1. Why Range-of-Motion and Trajectory Similarity Works

Although ROM similarity and landmark-trajectory similarity via DTW arise from different mathematical definitions, both metrics ultimately quantify a subject’s deviation from an ideal movement pattern. ROM similarity condenses the motion of a joint into a single amplitude measure—that is, the difference between observed and reference extremal angles—while DTW on joint trajectories evaluates the temporal alignment and shape of the entire movement cycle. In both cases, higher similarity implies closer adherence to the clinically guided exercise. By framing both ROM and DTW as proxies for “movement fidelity,” we can directly contrast our smartphone-based, markerless CV approach against QTM data, which clinicians already trust.

Selecting these two metrics was driven by our interdisciplinary team’s combined expertise and by the goal of demonstrating feasibility under real-world constraints. Our health-science collaborators are most comfortable interpreting ROM deviations, since that is standard in clinical motion-capture reports, whereas our AI team had experience implementing DTW for time-series analysis of pose-estimation outputs. By pairing established ROM benchmarks with trajectory-based DTW, we leveraged complementary strengths: ROM provided a simple, clinically interpretable score, and DTW captured the full spatiotemporal profile of each movement. This dual-metric strategy let us validate our markerless CV system both in terms that clinicians recognize and in the richer, sequence-level information that modern AI methods afford.

We acknowledge that a fully rigorous validation would compare identical variables—either both ROM measures or both trajectory measures—across systems. That deeper head-to-head comparison lies beyond this study’s scope. Instead, our proof-of-concept shows that low-cost, smartphone-based pose estimation can reproduce both the amplitude-based insights clinicians use (ROM) and the dynamic, cycle-by-cycle fidelity captured by DTW. Future work will extend this validation by collecting simultaneous IMU, video-only, and QTM data, applying a unified set of metrics to each.

#### 4.2.2. Evaluation Metrics

In order to evaluate such a system, we must first consider the task we are performing and what evaluation metrics better suit it. In this case, we are predicting a score for the quality of a given rehabilitation exercise based on measuring distance. In more practical terms, we are predicting a continuous value for each entry in our dataset, which, despite not being a regression problem, can be evaluated with typical regression metrics. With that in mind, we selected Mean Absolute Error (MAE), Root Mean Squared Error (RMSE), and the Pearson Correlation Coefficient (CC) to evaluate the proposed system. Although data were non-normally distributed, Pearson correlation was chosen for comparability with previous works. Future studies will incorporate non-parametric alternatives such as Spearman to capture non-linear relationships.

MAE measures the average absolute difference between the predicted scores and the actual scores. It provides an interpretable metric for understanding the overall accuracy of the proposed system’s predictions. Equation (8) shows how to calculate MAE, with *N* being the number of predictions, *y*_*i*_ being the actual values, and ${\hat{y}}_{i}$ being the predicted values.

(8) $$MAE=\frac{1}{N}\sum_{i=1}^{N}\left|y_{i}-{\hat{y}}_{i}\right|$$

RMSE calculates the square root of the average squared difference between the predicted scores and the actual scores. It penalizes larger errors more significantly than MAE and provides insight into the magnitude of prediction errors. Equation (9) is the RMSE equation, with *N*, *y*_*i*_, and ${\hat{y}}_{i}$ having the same meaning as in the previous section.

(9) $$RMSE=\sqrt{\frac{1}{N}\sum_{i=1}^{N}{\left(y_{i}-{\hat{y}}_{i}\right)}^{2}}$$

CC assesses the linear relationship between the predicted scores and the actual scores. It quantifies the strength and direction of the linear association, providing a measure of how well the variation in one variable explains the variation in another. In our case, it tells us how well the variation in the predicted scores explains the variation in the actual scores. Equation (10) presents the formula to compute CC, where *N*, *y*_*i*_, and ${\hat{y}}_{i}$ keep their meaning and *m* and $\hat{m}$ are the mean of *y* and $\hat{y}$, respectively.

(10) $$CC=\frac{\sum_{i=1}^{N}\left(y_{i}-m\right)\left({\hat{y}}_{i}-\hat{m}\right)}{\sqrt{\sum_{i=1}^{N}{\left(y_{i}-m\right)}^{2}\cdot \sum_{i=1}^{N}{\left({\hat{y}}_{i}-\hat{m}\right)}^{2}}}$$

#### 4.2.3. Outliers

Before we show the results obtained with the chosen metrics, mentioning outliers in our dataset is very important. As mentioned in Section 3.2, the dataset used in our work contains outliers that have been labeled as such by the researchers who collected the data. This means we have to handle them correctly, as they can have strong implications in our evaluation process.

One way to handle outliers is to remove them from the data directly [46], making it so that they would not affect our evaluation. However, our data is multidimensional, as we have a dimension for each joint group, Head, Trunk, and Shoulder, and the outliers appear only in one dimension. In the case of the *Diagonal* Exercise, the Head measurement of patient ID15 was considered an outlier. For the *Rotation* Exercise, the Head measurements of patients ID14 and ID15 were considered outliers. If we opt to remove the whole exercise from the dataset, we remove the outlier successfully, but we also remove valid data, more specifically, the measurements of the other joint groups of the mentioned patients.

Given this scenario, we could impute the value of the outliers. Imputation is a technique used to fill in missing data or handle outliers in a dataset by, for example, replacing them with the mean of the other values [47]. This would allow us to evaluate the complete dataset. Another alternative would be to remove the values just from the mentioned joint groups and evaluate the other joint groups for those patients. We ended up evaluating the joint groups separately, so it made sense to remove just those values instead of imputing them.

### 4.3. Results

After analyzing our data, normalizing it, and selecting the best metrics, we are ready to evaluate the proposed system. This section presents the results of the evaluation, in which the system’s performance is assessed through a comparison with the QTM. This comparison allows us to evaluate the proposed system’s efficacy against a very powerful tracking methodology. The results will be presented in two separate sections, one for each exercise. Each section has three subsections, one for each joint group. Each subsection includes scores normalized using Z-score normalization and the scores normalized using min-max scaling, as mentioned in Section 4.1.

#### 4.3.1. *Diagonal* Exercise

This section contains the results of the *Diagonal* exercise, divided into the three joint groups. For each joint group, we present the results obtained from the selected evaluation metrics: MAE, RMSE, and CC.

**Figure 12:**
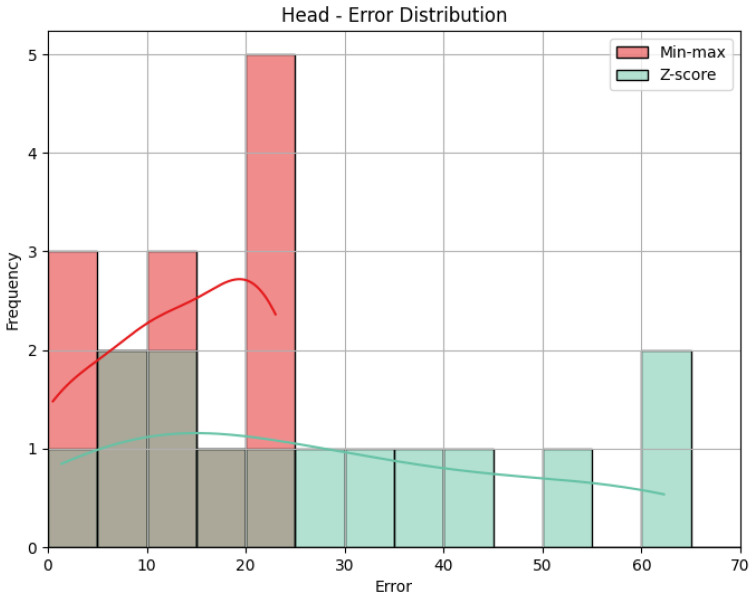
Error distribution for the Head joint group for the *Diagonal* exercise.

**Head**: The values obtained by the metrics for both the Head joint group of the *Diagonal* exercise are shown in Table 5, and Figure 12 shows the error distribution for the min-max predictions and the Z-score predictions.

Using min-max scaling, the Head joint group for the *Diagonal* exercise obtained an MAE of 13.29, an RMSE of 15.52, and a CC of 0.36. The value of the MAE tells us how big the errors were in our prediction, on average, in the scale of the results obtained, that is, 0 to 100. This means that the Head joint group’s predicted score was, on average, 13.29 points away from the score obtained by the QTM. The RMSE value calculates the difference between the squares of the errors, penalizing larger errors. In our case, the values are not too different, meaning that most errors are small and that there was little influence by large errors. The CC of 0.36 indicates a positive but weak correlation, as 0.36 is positive but small compared to the ideal value of 1. The interpretation of this value can be that the variation in our predictions is in the same direction as the actual values, meaning when our predictions increase, the actual values tend to increase as well, and vice-versa, but that relationship is not strong, meaning not much of the variation in the actual values can be explained by the variation in our predictions. When it comes to the Z-score results’ evaluation, if we compare the MAE and the RMSE to those of the min-max results, we can immediately detect that Z-score caused more errors, as the MAE is more than double, and larger errors, with an even larger difference of RMSE. This is expected, given what was explained in Section 4.1: Z-score normalization is not necessarily suitable for our data. The difference in errors can be visually understood with Figure 12. The CC is the same.

**Figure 13:**
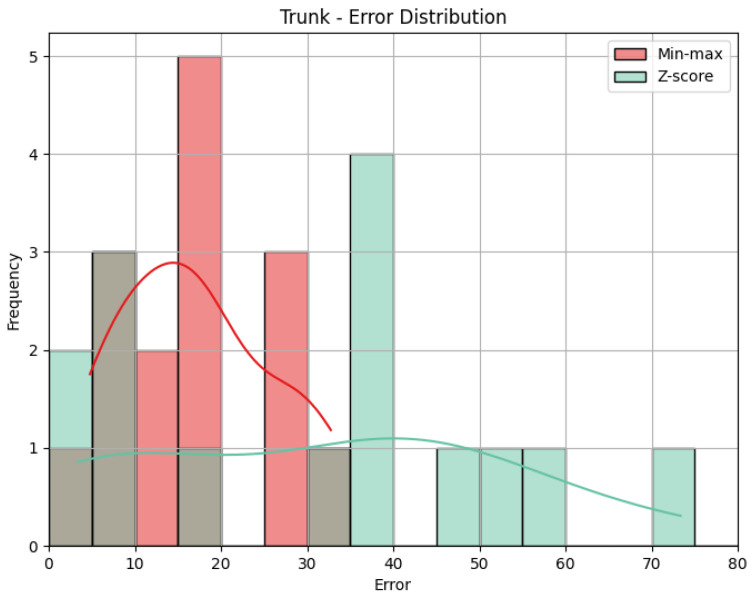
Error distribution for the Trunk joint group for the *Diagonal* exercise.

**Trunk**: The values obtained by the metrics for the Trunk joint group of the *Diagonal* exercise are shown in Table 6. In Figure 13, we can see the error distribution for both sets of predictions. With min-max scaling, the Trunk joint group obtained an MAE of 17.10, an RMSE of 19.12, and a CC of 0.47. The MAE of 17.10 reveals that, on average, the predicted scores for the Trunk joint group deviated by 17.10 points from the QTM scores. As for the Head, while there are some larger errors, the majority are smaller, as indicated by similar values of MAE and RMSE. The CC of 0.47 indicates a positive correlation, and it is relatively stronger than observed in the Head joint group. The variation in our predictions aligns with the variation in actual values, suggesting a reasonably strong directional relationship. In contrast, the Z-score results for the Trunk joint group show a higher MAE of 31.11 and a larger RMSE of 36.67 compared to min-max scaling. This notable increase in errors in magnitude and quantity reveals the difference between min-max and Z-score and can be better understood when we look at Figure 13. Again, the CC value is the same.

**Figure 14:**
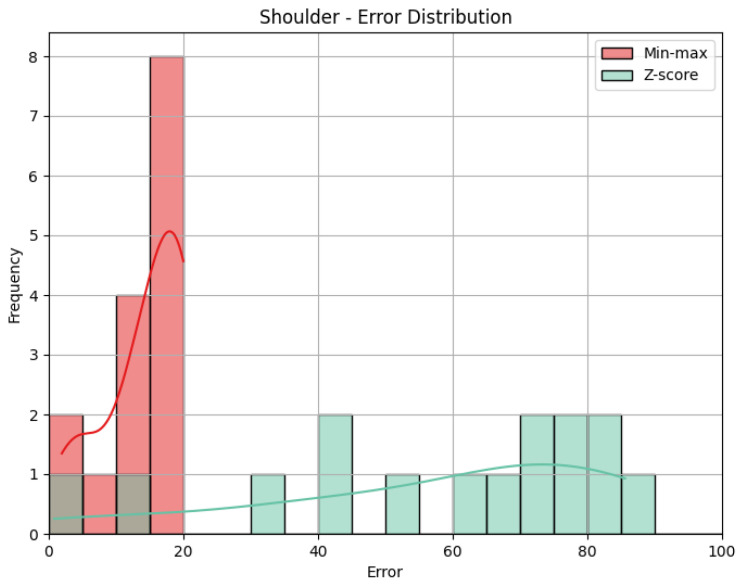
Error distribution for the Shoulder joint group for the *Diagonal* exercise.

**Shoulder**: The evaluation metrics for the Shoulder joint group during the *Diagonal* exercise are illustrated in Table 7, and the error distribution for both sets of predictions is represented in Figure 13. Under min-max scaling, the Shoulder joint group obtained an MAE of 14.44, an RMSE of 15.59, and a CC of −0.37. The MAE is similar to the one obtained by the Head and represents an average deviation of 14.44 points from the QTM scores. The values of MAE and RMSE suggest a reasonable level of accuracy, with few large errors, given how close the two values are. The CC of −0.37 indicates a negative correlation, which means that when the values in our predictions increase, the actual values decrease, which is not ideal. However, this correlation is weak, which means not much of the variation in the actual values can be explained by the variation in our predictions. Following the trend of the other two joint groups, the Z-score results for the Shoulder joint group show a substantially higher MAE of 57.59 and a larger RMSE of 62.97. In this case, the difference is even greater, meaning a considerable increase in errors, as can be seen in Figure 14.

#### 4.3.2. *Rotation* Exercise

This section contains the results of the Rotation exercise, divided into the three joint groups. For each joint group, we present the results obtained from the selected evaluation metrics: MAE, RMSE, and CC.

**Figure 15:**
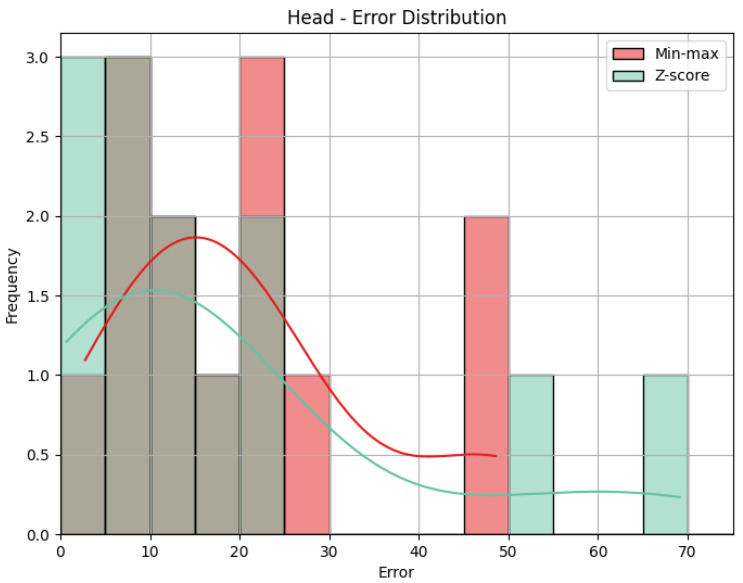
Error distribution for the Head joint group for the *Rotation* exercise.

**Head**: The values obtained by the metrics for the Head joint group of the *Rotation* exercise are shown in Table 8. Figure 15 shows the error distribution for the Head joint group during the *Rotation* exercise. The MAE of 20.41 implies that, on average, the predicted scores for the Head joint group deviated by 20.41 points from the QTM scores. The RMSE, measuring the square root of the average squared errors, is slightly larger, indicating the presence of some large errors. The CC of 0.22 indicates a positive but weak correlation. This suggests a directional relationship between our predictions and the actual values, but the correlation strength is low. Utilizing Z-score normalization, the Head joint group achieved an MAE of 18.86 and an RMSE of 27.11. While the MAE shows a slight improvement in prediction accuracy compared to min-max scaling, the RMSE reflects an increase in the magnitude of errors. In Figure 15, we can see that Z-score shows, in general, smaller errors than min-max, explaining the lower MAE, but, as we can see, it also shows one very large error, penalized by RMSE. As mentioned in the previous sections, the CC is the same for Z-score and min-max results.

**Figure 16:**
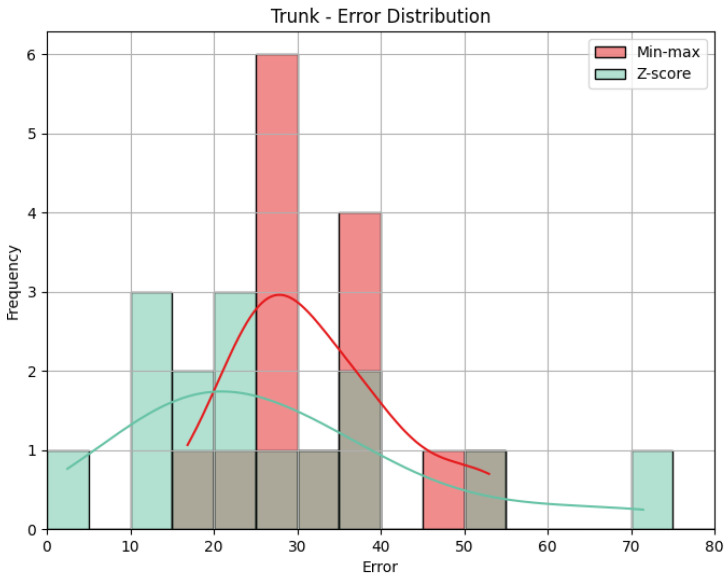
Error distribution for the Trunk joint group for the *Rotation* exercise.

**Trunk**: Table 9 displays the evaluation metrics for the Trunk joint group during the *Rotation* exercise, and in Figure 16, we can see the error distribution for the Trunk joint group during the *Rotation* exercise. With min-max scaling, the Trunk joint group achieved an MAE of 32.30, an RMSE of 33.69, and a CC of 0.51. The MAE of 32.30 is the largest of any joint group with min-max, indicating larger errors on average. A similar value for RMSE reveals that the largest error must not be much larger than the other errors. However, because the MAE is already large, the RMSE could still indicate the presence of large errors. The CC of 0.51 reveals a positive correlation with a moderate strength. This suggests a reasonably strong directional relationship between our predictions and the actual values for the Trunk joint group. Switching to Z-score normalization, the Trunk joint group achieved an MAE of 27.30 and an RMSE of 32.25. Notably, Z-score normalization resulted in a decrease in the MAE, indicating an improvement in prediction accuracy, while the value of the RMSE being close to the one obtained with the min-max results implies a comparable distribution of errors. Figure 16 shows that Z-score presents many more errors close to 0, but there are still some very large errors, while min-max’s errors are generally larger. Once more, the CC is the same.

**Figure 17:**
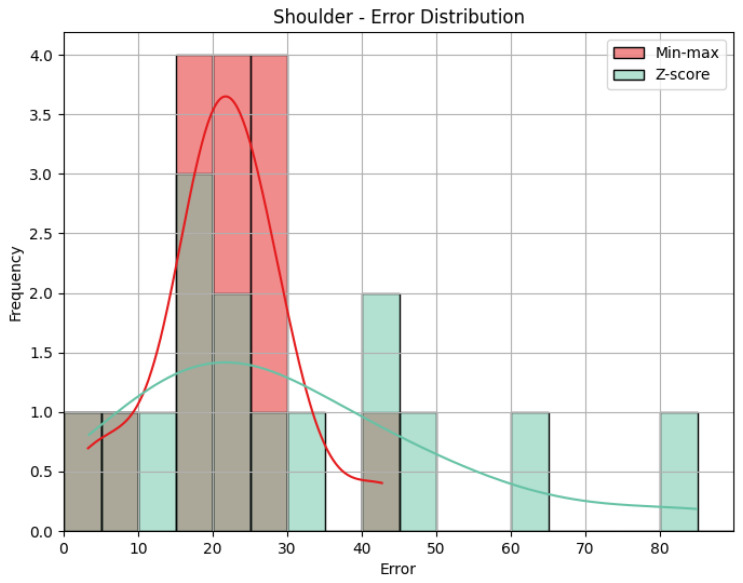
Error distribution for the Shoulder joint group for the *Rotation* exercise.

**Shoulder**: The evaluation metrics for the Shoulder joint group during the ’Rotation’ exercise are illustrated in Table 10. Figure 17 displays the error distribution for the Trunk joint group during the *Rotation* exercise. When employing min-max scaling, the Shoulder joint group obtained an MAE of 21.45, an RMSE of 23.19, and a CC of −0.37. Once again, the value of the MAE indicates that, on average, the predicted scores for the Shoulder joint group deviated by 21.45 points from the QTM scores. The RMSE suggests reasonably large errors, as it is larger than MAE. As for the Shoulder group of the *Diagonal* exercise, the CC of −0.37 indicates a negative correlation, revealing an inverse relationship between our predictions and the actual values for the Shoulder joint group. However, this correlation is weak, with a relatively low magnitude. Upon employing Z-score normalization, the Shoulder joint group achieved an MAE of 30.30 and an RMSE of 36.83. Z-score normalization resulted in an increase in both the MAE, indicating a decrease in prediction accuracy, and the RMSE, revealing the presence of larger errors. Figure 17 clarifies the difference between MAE and RMSE values. Min-max shows not only smaller values on average but also larger values that are smaller than the ones obtained by Z-score. Once again, the value of the CC is not affected by normalization.

### 4.4. Results Discussion

The presented results shed light on the performance of the proposed system in predicting the quality scores of rehabilitation exercises compared to the QTM. In this section, we discuss key findings and potential implications of the obtained results. Before we take a longer look at the results, however, it is relevant to state that the choice of normalization method significantly impacts the results obtained. Min-max scaling consistently outperforms Z-score normalization, showcasing its suitability for our dataset, as we had clear minimum and maximum values. While Z-score normalization is widely adopted, our findings emphasize the importance of exploring alternative normalization techniques, as suggested by Lima and Souza [45]. It is also important to mention outlier handling and how crucial it is. Our decision was to remove values from specific joint groups rather than entire exercises, as this approach preserves valuable data while addressing the influence of outliers on specific joint measurements.

#### 4.4.1. Overall Performance Trends

Across both the *Diagonal* and *Rotation* exercises, the system demonstrates varying levels of accuracy and correlation for different joint groups. That is noticeable when we look at the ranges of values obtained for each calculated error metric. When it comes to the min-max scores, MAE ranged from 13.29 to 32.30, and RMSE ranged from 15.52 to 33.69. In both metrics, a difference of more than double is present. Z-score MAE ranged from 18.86 to 57.7, and RMSE ranged from 27.11 to 62.97. This tells us that performances were not consistent over different joint groups and exercises. In terms of correlation, we obtained reasonable results overall, with most of the correlations being positive, including the Trunk group, which obtained CCs around the 0.5 mark. However, the Shoulder group evaluation resulted in negative coefficients, which indicates an inverse relationship between the predicted and the actual values. This means that, overall, there was a mixture of results, which means we must compare results between exercises and joint groups and discuss whether there are any indications of one exercise outperforming the other or one joint group outperforming the others.

#### 4.4.2. Exercise-Specific Observations

This subsection includes observations for each exercise and joint group, allowing for comparison between them and a better understanding of the results obtained.

***Diagonal* Exercise** Considering the min-max results for the *Diagonal* exercise, the Head group presents modest errors and exhibits a positive correlation, which, despite being weak, suggests a consistent directional relationship. The Trunk group’s errors are slightly larger and include a positive correlation, highlighting reasonable prediction accuracy. The Shoulder groups’ evaluation metrics’ results are between those of the Head and the Trunk groups. However, a negative correlation suggests that predictions may not be related to actual values. Overall, the min-max scores for this exercise obtained reasonable results, with an average MAE of 14.94 and an average RMSE of 16.74. Although the Head had fewer errors, the values are too close to state a clear difference between any of the joint groups. With the Z-score scores, the errors were larger. While the Head and Trunk groups obtained MAE and RMSE values close to 30, which would already be worse than the min-max scores, the Shoulder group obtained an MAE and an RMSE close to 60, revealing that the average prediction was off by almost 60 points.
***Rotation* Exercise** Regarding the min-max results for the *Rotation* exercise, the Head and Shoulder groups obtained similar error metrics, and the Trunk group obtained a higher degree of error. In terms of correlation, the trend from the *Diagonal* exercise continues, with the Head obtaining a weak positive correlation, the Trunk a reasonably high positive correlation, and the Shoulder a weak negative correlation. Compared with the *Diagonal* exercise, the min-max scores for this exercise obtained worse results, with an average MAE of 24.72 and an average RMSE of 27.16, practically 10 points higher than the respective metrics for the *Diagonal* exercise. Interestingly, the Z-score scores obtained very similar, if not better, performances to those of the min-max scores in both the Head and the Trunk groups, obtaining slightly lower MAEs and similar RMSEs.

#### 4.4.3. Discussion Summary

In summary, the evaluation of the rehabilitation exercise prediction system offers key insights into its performance across the *Diagonal* and *Rotation* exercises, focusing on joint groups – Head, Trunk, and Shoulder. The results highlight the impact of data normalization, emphasizing the superior performance of min-max scaling over Z-score normalization. The Shoulder joint group showed weaker correlation, likely due to occlusion, depth estimation errors, or limited reliability in upper-body tracking during seated exercises. And while the current implementation uses uniform joint weighting in DTW, future extensions may incorporate adaptive or learned weighting schemes to prioritize clinically relevant joints and improve sensitivity to movement nuances. The chosen evaluation metrics—MAE, RMSE, and CC—help us understand the accuracy of the performance of the proposed system and how related our predictions may be with the actual scores, unveiling performance trends across joint groups and exercises, such as the overall better performance of the proposed system during the *Diagonal* exercise and the higher correlation results yielded from the Trunk group. Although there is currently no established clinical threshold for these metrics in rehabilitation, preliminary discussions with clinicians from Escola de Saúde suggest this level of deviation may be tolerable for at-home progress monitoring. Our vision-based and the Qualisys scores differ by at most ∼0.1 units (with *p* ≤ 0.001). These very small differences indicate high agreement with the (at the moment) gold-standard system. However, and while this system shows promising alignment with a gold-standard QTM assessment, it is not yet clinically validated for deployment. As this was a pilot study with only two exercises and 15 participants, the results may not generalize across populations, body types, or rehabilitation protocols. Future validation will require larger, more diverse cohorts and task variety to mitigate the risk of overfitting.

### 4.5. Statistical Analysis

After computing traditional evaluation metrics, we wanted to better understand the results obtained through hypothesis testing. Given the nature of our data, characterized by a relatively small population and the inability to assume a specific distribution, we opted for non-parametric tests. More specifically, we opted for the Wilcoxon Signed-Rank Test [48], which is particularly suitable for paired samples, in our case the predicted and actual scores, providing a robust analysis of whether there is a significant difference between them.

#### Wilcoxon Signed-Rank Test

The Wilcoxon Signed-Rank Test [48] is a non-parametric statistical test used to assess whether there is a significant difference between paired samples. The basic idea is to rank the absolute differences between the pairs of observations, which in our case are the errors, ignore the signs, and sum the ranks of the positive or negative differences. The null hypothesis assumes that the distribution of these differences is symmetric around zero. The test statistic is then calculated based on these ranks [49]. The steps for performing this test are as follows:

1. For each pair of observations, calculate the absolute difference between the predicted and the actual score.
2. Rank these absolute differences from smallest to largest, ignoring the signs.
3. Taking the signs back into account, sum all the positive ranks (*R*_−_) and all the negative ranks (*R*_+_).
4. Calculate the smallest value between *R*_−_ and *R*_+_: *R* = min(*R*_−_, *R*_+_).

The final value, *R*, is the test statistic and can be compared with tabled critical values to obtain the *p*-value. That *p*-value is used to assess the significance of the difference found between the two sets of scores. If it is under a given significance value, commonly 0.05, we can reject the null hypothesis and state that there is a significant statistical difference between the two sets of scores. If it is over that value, we cannot confirm that that statistical difference is significant. Thus, we performed the Wilcoxon Signed-Rank Test for the differences between the min-max scores and the actual scores and the differences between the Z-score scores and the actual scores, divided into the three joint groups. We performed the test once for each exercise. Table 11 presents the results for the Wilcoxon Signed-Rank Test for the *Rotation* exercise.

**Figure 18:**
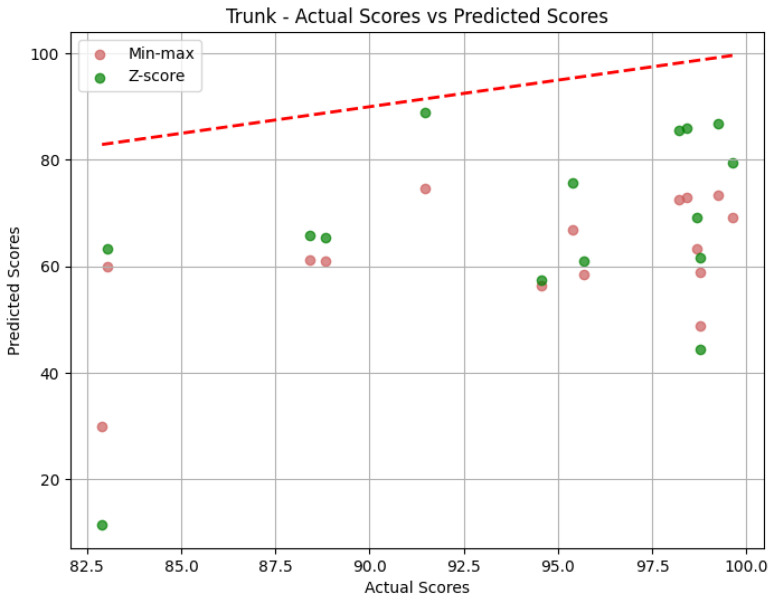
Scatter plot of predictions for the Trunk joint group for the *Rotation* exercise.

**Figure 19:**
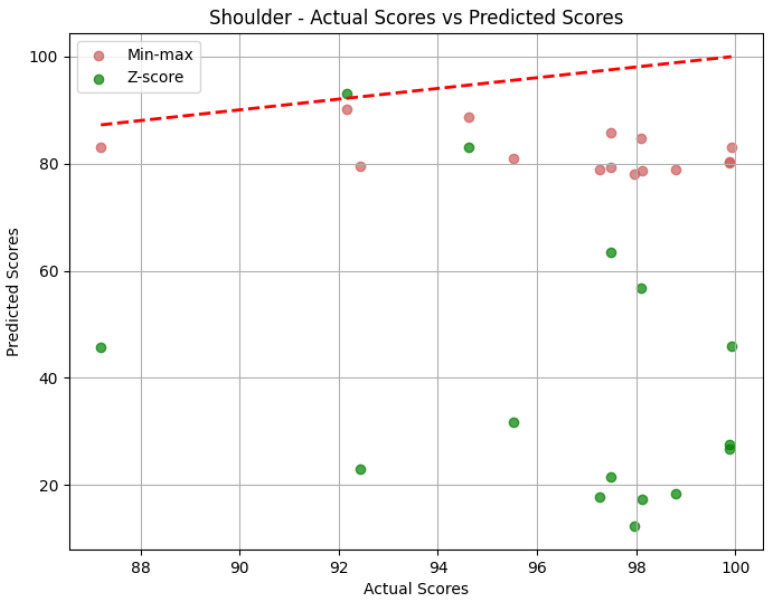
Scatter plot of predictions for the Shoulder joint group for the *Diagonal* exercise.

One problem with this test was noticed immediately, as the P-values of the Trunk joint group of the *Rotation* exercise for both the min-max and the Z-score scores are 0, as well as the P-value of the Shoulder group of the *Diagonal* exercise for the min-max scores. This is due to the fact that, for those particular scores, all of the predictions were lower than the actual score. This can be confirmed by interpreting Figure 18 and Figure 19, which display a scatter plot of the min-max and Z-score predictions against the actual scores.

As we can see, all the predictions in Figure 18 are under the red dashed line that indicates a correct prediction, meaning that all the differences are negative. The same thing happens for the min-max predictions in Figure 19.

Going back to how the Wilcoxon Signed-Rank Test works, it is easy to understand that if the differences are all positive or all negative, one of *R*_−_ or *R*_+_ will be 0, and thus *R* will always be 0. In these cases, the Wilcoxon Signed-Rank Test does not provide meaningful information. However, the other values are of interest, and we can interpret them following what was mentioned earlier. For the *Diagonal* exercise, the min-max Head value is more than 0.05, indicating that, for that pair of sets of scores, we cannot reject the null hypothesis. That is, we cannot confirm that there is a significant statistical difference between the sets of scores that generated those *p*-values. The rest of the sets of scores had *p*-values less than 0.05, confirming that the difference between the paired sets is not by chance and holds statistical significance.

For the Shoulder min-max value, which is less than 0.05, we reject the null hypothesis and confirm the statistical significance of the differences between the sets of scores.

An important note: no correction for multiple comparisons was applied, so *p*-values should be interpreted cautiously. Future work will adopt stricter controls such as Bonferroni correction to reduce Type I error risk.

### 4.6. Summary

This section focused on evaluating our CV-based motion tracking system for rehabilitation. We began by discussing the importance of data normalization, specifically for comparing results obtained from a distance-based system with a ground truth dataset labeled on a 0 to 100 scale. Three normalization methods were presented: Z-score normalization, min-max scaling, and maximum absolute scaling. We mentioned that we opted to use min-max scaling, based on the characteristics of the dataset, but also Z-score normalization, as it is very commonly used. We then explored the system evaluation process, emphasizing the significance of choosing appropriate metrics. MAE, RMSE, and CC were selected to assess the system’s prediction accuracy. We also addressed the outliers in the dataset and how we handled them by removing specific joint group measurements rather than entire exercises. Then, we presented the results for the *Diagonal* and *Rotation* exercises, focusing on the Head, Trunk, and Shoulder joint groups. We discussed the results, highlighting the impact of data normalization and how min-max scaling outperformed Z-score normalization for the most part. We touched on overall performance trends that showed varying levels of accuracy across the different exercises and joint groups. Thus, we included exercise-specific observations, revealing that the system performed better during the *Diagonal* exercise and that the Trunk joint group obtained higher overall correlation results. After evaluation, we employed hypothesis testing to gain insights into the evaluation metrics obtained earlier. We introduced parametric and non-parametric tests and explained the difference between the two. In our case, there was a preference for non-parametric methods due to the dataset’s characteristics, such as a small population and the absence of assumptions about data distribution. The Wilcoxon Signed-Rank Test was specifically chosen for its applicability to paired samples, comparing predicted and actual scores. The test was explained, and the steps for performing it were outlined. However, a limitation with the test arose, as when all predictions are consistently lower than actual scores, the test is rendered ineffective. Despite this limitation, the section provides valuable insights into the statistical significance of differences between predicted and actual scores for various exercise and joint group combinations.

## 5. Conclusions

In this paper, we have explored the fields of Computer Vision systems and rehabilitation and how the former can be applied to improve the latter and make it more accessible. Our main goal was to develop a CV-based system that allows patients to perform rehabilitation exercises independently, using accessible technology, while receiving necessary and adequate feedback. In order to do that, we had to research the two main fields involved: Rehabilitation and Computer Vision.

This study presents a preliminary but promising comparison between a smartphone-based, markerless pose estimation system and a gold-standard QTM motion analysis. Although the accuracy metrics are not yet sufficient for full clinical substitution, they are within the range of acceptable error margins for certain rehabilitation contexts. This supports the hypothesis that such systems can provide useful patient feedback or augment remote clinical supervision, particularly where access to expensive motion capture setups is limited. This study lays the groundwork for accessible, data-driven rehabilitation support, paving the way for future clinical trials and system deployment at scale.

### 5.1. Summary of Main Takeaways

In the course of this research, we made pivotal observations and conclusions, from which we want to highlight the most important ones. Firstly, we reviewed the current state of the art in Computer Vision systems for assessing physical rehabilitation exercises. Then, we successfully built a CV-based motion tracking system while prioritizing accessibility. That means we successfully demonstrated that there is accessible technology currently available to fill this gap. This could indicate a shift toward developing rehabilitation systems based on accessible technology. One of the aspects of our work that greatly supports that shift is the fact that the smartphone can be used to successfully capture rehabilitation-relevant data. As has been mentioned before, we agree with Lam et al. [24] that smartphones will play a pivotal role in the future of rehabilitation. Another important takeaway is that DTW can be used to compare two videos. Despite the trend being the integration of Deep Learning, we further validate in our research that DTW can be used as a benchmark. Finally, we also confirm that Z-score normalization does not suit non-normally distributed data. Our findings underscore the significance of choosing appropriate normalization methods, with min-max scaling emerging as a preferred alternative in scenarios where there is a clear maximum and minimum defined. These takeaways encapsulate the key achievements and insights derived from our research, serving as a brief summary of what can be concluded from this work.

### 5.2. Opportunities for Improvement and Future Work

The final result of this paper can still be improved, which is shown by the results being worse than desired. One of the main opportunities for improvement is the lack of exploration of alternatives for the *Comparison and Assessment* component of the proposed system. More specifically, as understood from Section 2, while DTW shows interesting results, the trend is to explore Deep Learning. Moving forward, this should be the main focus of future work. Its integration into the project was hindered by time constraints and the fact that the dataset available was not particularly big. We also believe that conducting an intraclass correlation analysis to assess the agreement between the QTM and mediapipe/DTW methods would be beneficial. This approach could yield more appropriate and interpretable results.

At present, our study establishes a foundational proof-of-concept, demonstrating that low-cost, smartphone-based pose estimation can approximate both clinicians’ traditional ROM assessments and the richer, sequence-level fidelity captured by DTW. Building on this groundwork, future work should pursue a unified validation framework in which identical metrics are computed from synchronized QTM, smartphone video, and IMU recordings. Such a head-to-head design would eliminate questions about cross-metric correspondence and provide a clearer picture of how markerless CV stacks up against each gold-standard modality. This effort could also explore hybrid metrics that blend angle extrema with temporal alignment scores, potentially yielding an even more robust movement-quality index.

Beyond metric unification, there are opportunities to broaden the system’s applicability and resilience. Integrating real-time feedback loops—where the smartphone app not only analyzes but also guides patients through corrective cues—would transform the tool from a passive recorder into an active rehabilitation coach. Expanding the study population to include individuals with diverse movement impairments and across multiple exercise types would further test the generalizability of our approach. Finally, exploring emerging lightweight neural architectures for on-device inference could reduce reliance on cloud processing, enhancing privacy and minimizing latency for truly ubiquitous, at-home rehabilitation support.

Another aspect to consider is the ethical outlines of this work. In a possible integration in an application made available to patients, a comprehensive exploration of ethical guidelines should be performed, prioritizing patient privacy. This could include collaboration with professionals to align technological advancement with ethical considerations. Efforts should be made to keep the patients safe. Acknowledging limitations serves as a stepping stone for future advancements. The outlined areas for future work preview how the system could be improved, aligning it with research trends and upholding ethical standards.

## Figures and Tables

**Table 1:** Scientific articles on motion tracking systems for rehabilitation.

Article	Data Acquisition	Feature Engineering	Comparison and Assessment
[8]	Microsoft Kinect	OpenNI	DTW
[9]	Microsoft Kinect	Kinect SDK	ANFIS for performance and speed components and Fuzzy Logic to combine those into a score
[10]	Microsoft Kinect	Kinect SDK	HSMM
[11]	Microsoft Kinect	Kinect SDK	N/A
[12]	Microsoft Kinect	Kinect SDK	N/A
[13]	RGB Camera	N/A	Custom scoring function and Fuzzy Logic to obtain an overall score
[14]	RGB Camera	OpenPose	Modular NN
[15]	RGB Camera	PoseNet	Not Specified
[16]	RGB Camera	BlazePose	N/A
[17]	RGB Camera	OpenFace	N/A
[18]	RGB Camera	OpenPose	N/A
[19]	RGB Camera	Convolutional Pose Machines	N/A
[20]	N/A	N/A	Deep NN and GMM log-likelihood performance metric

**Table 2:** Relevant datasets for CV-based rehabilitation.

Dataset	Number of Individuals	Number of Exercises
IntelliRehabDS [33]	29	9
UI-PRMD [34]	10	10
KIMORE [35]	78	5

**Table 3:** First 15 rows of the distances file for the *Diagonal* exercise.

ID	Head	Trunk	Shoulder
ID01_1	220.63	50.39	103.13
ID01_2	258.58	55.52	109.38
ID01_3	257.50	55.06	122.51
ID02_1	272.44	37.97	72.85
ID02_2	212.43	32.74	80.76
ID02_3	310.14	47.60	89.19
ID03_1	159.56	32.30	99.23
ID03_2	152.75	29.36	102.72
ID03_3	176.59	31.23	86.32
ID04_1	235.18	54.42	82.61
ID04_2	188.21	47.61	90.15
ID04_3	203.35	60.69	86.92
ID05_1	308.34	70.13	117.46
ID05_2	301.78	67.27	106.83
ID05_3	316.42	65.68	113.23

**Table 4:** Scores file for the *Rotation* exercise using min-max scaling.

ID	Head	Trunk	Shoulder
ID01	60.08	56.45	77.31
ID02	55.95	61.03	76.73
ID03	80.08	72.93	76.14
ID04	65.33	58.81	76.47
ID05	63.32	61.25	80.64
ID06	72.1	73.42	79.74
ID07	70.18	63.21	67.68
ID08	73.92	69.19	65.31
ID09	69.99	66.98	80.01
ID10	57.75	59.85	78.93
ID11	70.94	74.62	78.15
ID12	29.1	29.92	54.14
ID13	65.54	58.5	70.37
ID14	78.75	72.59	82.31
ID15	62.81	48.89	59.19

**Table 5:** Evaluation metrics for the Head joint group for the *Diagonal* exercise.

Normalization	MAE	RMSE	CC
Min-max	13.29	15.52	0.36
Z-score	27.88	34.36	

**Table 6:** Evaluation metrics for the Trunk joint group for the *Diagonal* exercise.

Normalization	MAE	RMSE	CC
Min-max	17.10	19.12	0.47
Z-score	31.11	37.67	

**Table 7:** Evaluation metrics for the Shoulder joint group for the *Diagonal* exercise.

Normalization	MAE	RMSE	CC
Min-max	14.44	15.59	−0.37
Z-score	57.59	62.97	

**Table 8:** Evaluation metrics for the Head joint group for the *Rotation* exercise.

Normalization	MAE	RMSE	CC
Min-max	20.41	24.60	0.22
Z-score	18.86	27.11	

**Table 9:** Evaluation metrics for the Trunk joint group for the *Rotation* exercise.

Normalization	MAE	RMSE	CC
min-max	32.30	33.69	0.51
Z-score	27.30	32.25	

**Table 10:** Evaluation metrics for the Shoulder joint group for the *Rotation* exercise.

Normalization	MAE	RMSE	CC
Min-max	21.45	23.19	−0.37
Z-score	30.30	36.83	

**Table 11:** Wilcoxon Signed-Rank Test results for min-max and Z-score scores for the *Rotation* and *Diagonal* exercises.

Exercise	Joint Group	*p*-Value Min-Max	*p*-Value Z-Score
Diagonal	Head	0.057	0.005
	Trunk	0.001	0.002
	Shoulder	0.000	0.000
Rotation	Head	0.057	0.334
	Trunk	0.000	0.000
	Shoulder	0.035	0.107

## Data Availability

Data are contained within the article.

## References

[1] Machlin S., Chevan J., Yu W., Zodet M. (2011). Determinants of Utilization and Expenditures for Episodes of Ambulatory Physical Therapy Among Adults. Phys. Ther..

[2] Mehmood F., Mumtaz N., Mehmood A. (2025). Next-Generation Tools for Patient Care and Rehabilitation: A Review of Modern Innovations. Actuators.

[3] Lopes M., Melo A.S.C., Cunha B., Sousa A.S.P. (2023). Smartphone-Based Video Analysis for Guiding Shoulder Therapeutic Exercises: Concurrent Validity for Movement Quality Control. Appl. Sci..

[4] Colyer S.L., Evans M., Cosker D.P., Salo A.I.T. (2018). A Review of the Evolution of Vision-Based Motion Analysis and the Integration of Advanced Computer Vision Methods Towards Developing a Markerless System. Sport. Med.-Open.

[5] Hellsten T., Karlsson J., Shamsuzzaman M., Pulkkis G. (2021). The Potential of Computer Vision-Based Marker-Less Human Motion Analysis for Rehabilitation. Rehabil. Process Outcome.

[6] Lobo P., Morais P., Murray P., Vilaça J.L. (2024). Trends and Innovations in Wearable Technology for Motor Rehabilitation, Prediction, and Monitoring: A Comprehensive Review. Sensors.

[7] Debnath B., O’Brien M., Yamaguchi M., Behera A. (2022). A review of computer vision-based approaches for physical rehabilitation and assessment. Multimed. Syst..

[8] Chen Y.L., Liu C.H., Yu C.W., Lee P., Kuo Y.W. (2018). An Upper Extremity Rehabilitation System Using Efficient Vision-Based Action Identification Techniques. Appl. Sci..

[9] Su C.J., Chiang C.Y., Huang J.Y. (2014). Kinect-enabled home-based rehabilitation system using Dynamic Time Warping and fuzzy logic. Appl. Soft Comput..

[10] Capecci M., Ceravolo M.G., Ferracuti F., Iarlori S., Kyrki V., Monteriù A., Romeo L., Verdini F. (2018). A Hidden Semi-Markov Model based approach for rehabilitation exercise assessment. J. Biomed. Inform..

[11] Dorado J., del Toro Garcia X., Santofimia M., Parreño-Torres A., Cantarero R., Rubio Ruiz A., López J.C. (2019). A computer-vision-based system for at-home rheumatoid arthritis rehabilitation. Int. J. Distrib. Sens. Netw..

[12] Zhi Y.X., Lukasik M., Li M.H., Dolatabadi E., Wang R.H., Taati B. (2018). Automatic Detection of Compensation During Robotic Stroke Rehabilitation Therapy. IEEE J. Transl. Eng. Health Med..

[13] Ciabattoni L., Ferracuti F., Iarlori S., Longhi S., Romeo L. A novel computer vision based e-rehabilitation system: From gaming to therapy support. Proceedings of the 2016 IEEE International Conference on Consumer Electronics (ICCE).

[14] Francisco J.A., Rodrigues P.S. (2022). Computer Vision Based on a Modular Neural Network for Automatic Assessment of Physical Therapy Rehabilitation Activities. IEEE Trans. Neural Syst. Rehabil. Eng..

[15] Leechaikul N., Charoenseang S. (2021). Computer Vision Based Rehabilitation Assistant System.

[16] Yang H., Wang Y., Shi Y. Rehabilitation Training Evaluation and Correction System Based on BlazePose. Proceedings of the 2022 IEEE 4th Eurasia Conference on IOT, Communication and Engineering (ECICE).

[17] Abbas A., Yadav V., Smith E., Ramjas E., Rutter S., Benavidez C., Koesmahargyo V., Zhang L., Guan L., Rosenfield P. (2021). Computer Vision-Based Assessment of Motor Functioning in Schizophrenia: Use of Smartphones for Remote Measurement of Schizophrenia Symptomatology. Digit. Biomark..

[18] Ferrer-Mallol E., Matthews C., Stoodley M., Gaeta A., George E., Reuben E., Johnson A., Davies E.H. (2022). Patient-led development of digital endpoints and the use of computer vision analysis in assessment of motor function in rare diseases. Front. Pharmacol..

[19] Li M.H., Mestre T.A., Fox S.H., Taati B. (2018). Vision-based assessment of parkinsonism and levodopa-induced dyskinesia with pose estimation. J. NeuroEng. Rehabil..

[20] Liao Y., Vakanski A., Xian M. (2020). A Deep Learning Framework for Assessing Physical Rehabilitation Exercises. IEEE Trans. Neural Syst. Rehabil. Eng..

[21] Mousavi Hondori H., Khademi M. (2014). A Review on Technical and Clinical Impact of Microsoft Kinect on Physical Therapy and Rehabilitation. J. Med Eng..

[22] Microsoft (2022). Kinect. https://learn.microsoft.com/en-us/windows/apps/design/devices/kinect-for-windows.

[23] Mourcou Q., Fleury A., Diot B., Franco C., Vuillerme N. (2015). Mobile Phone-Based Joint Angle Measurement for Functional Assessment and Rehabilitation of Proprioception. BioMed Res. Int..

[24] Lam W.W.T., Tang Y.M., Fong K.N.K. (2023). A systematic review of the applications of markerless motion capture (MMC) technology for clinical measurement in rehabilitation. J. NeuroEng. Rehabil..

[25] Cao Z., Hidalgo G., Simon T., Wei S.E., Sheikh Y. (2019). OpenPose: Realtime Multi-Person 2D Pose Estimation using Part Affinity Fields. arXiv.

[26] Bazarevsky V., Grishchenko I., Raveendran K., Zhu T., Zhang F., Grundmann M. (2020). BlazePose: On-device Real-time Body Pose tracking. arXiv.

[27] Falahati S. (2013). OpenNI Cookbook.

[28] Kendall A., Grimes M., Cipolla R. (2016). PoseNet: A Convolutional Network for Real-Time 6-DOF Camera Relocalization. arXiv.

[29] Wei S.E., Ramakrishna V., Kanade T., Sheikh Y. (2016). Convolutional Pose Machines. arXiv.

[30] Baltrusaitis T., Robinson P., Morency L.P. (2016). OpenFace: An open source facial behavior analysis toolkit. Proceedings of the 2016 IEEE Winter Conference on Applications of Computer Vision (WACV).

[31] Exer (2023). Exer Health. https://www.exer.ai/product/health.

[32] Pereira B., Cunha B., Viana P., Lopes M., Melo A.S.C., Sousa A.S.P. (2024). A Machine Learning App for Monitoring Physical Therapy at Home. Sensors.

[33] Miron A., Sadawi N., Ismail W., Hussain H., Grosan C. (2021). IntelliRehabDS (IRDS)—A Dataset of Physical Rehabilitation Movements. Data.

[34] Vakanski A., Jun H.P., Paul D., Baker R. (2018). A Data Set of Human Body Movements for Physical Rehabilitation Exercises. Data.

[35] Capecci M., Ceravolo M.G., Ferracuti F., Iarlori S., Monteriù A., Romeo L., Verdini F. (2019). The KIMORE Dataset: KInematic Assessment of MOvement and Clinical Scores for Remote Monitoring of Physical REhabilitation. IEEE Trans. Neural Syst. Rehabil. Eng..

[36] Google (2023). MediaPipe. https://developers.google.com/mediapipe.

[37] Lugaresi C., Tang J., Nash H., McClanahan C., Uboweja E., Hays M., Zhang F., Chang C.L., Yong M., Lee J. MediaPipe: A Framework for Perceiving and Processing Reality. Proceedings of the Third Workshop on Computer Vision for AR/VR at IEEE Computer Vision and Pattern Recognition (CVPR) 2019.

[38] Google (2023). Pose Landmark Detection Guide. https://developers.google.com/mediapipe/solutions/vision/pose_landmarker.

[39] Sakoe H., Chiba S. (1978). Dynamic programming algorithm optimization for spoken word recognition. IEEE Trans. Acoust. Speech Signal Process..

[40] Kulkarni N. (2017). Effect of Dynamic Time Warping using different Distance Measures on Time Series Classification. Int. J. Comput. Appl..

[41] Qualisys A. (2006). Qualisys Track Manager User Manual.

[42] Bradski G.R., Kaehler A. (2000). OpenCV. Dr. Dobb’s J. Softw. Tools.

[43] Wu R., Keogh E. (2020). FastDTW is Approximate and Generally Slower Than the Algorithm it Approximates. IEEE Trans. Knowl. Data Eng..

[44] Salvador S., Chan P. FastDTW: Toward accurate dynamic time warping in linear time and space. Proceedings of the KDD Workshop on Mining Temporal and Sequential Data.

[45] Lima F.T., Souza V.M.A. (2023). A Large Comparison of Normalization Methods on Time Series. Big Data Res..

[46] Aguinis H., Gottfredson R.K., Joo H. (2013). Best-Practice Recommendations for Defining, Identifying, and Handling Outliers. Organ. Res. Methods.

[47] Chambers R.L., Ren R. (2004). Chambers, R.L.; Ren, R. Outlier robust imputation of survey data. Proc. Am. Stat. Assoc..

[48] Wilcoxon F. (1945). Individual Comparisons by Ranking Methods. Biom. Bull..

[49] Whitley E., Ball J. (2002). Statistics review 6: Nonparametric methods. Crit. Care.

